# Protein Phosphatase 1 β Paralogs Encode the Zebrafish Myosin Phosphatase Catalytic Subunit

**DOI:** 10.1371/journal.pone.0075766

**Published:** 2013-09-11

**Authors:** Vaishali Jayashankar, Michael J. Nguyen, Brandon W. Carr, Dale C. Zheng, Joseph B. Rosales, Joshua B. Rosales, Douglas C. Weiser

**Affiliations:** Department of Biological Sciences, University of the Pacific, Stockton, California, United States of America; Western University, Canada

## Abstract

**Background:**

The myosin phosphatase is a highly conserved regulator of actomyosin contractility. Zebrafish has emerged as an ideal model system to study the *in* *vivo* role of myosin phosphatase in controlling cell contractility, cell movement and epithelial biology. Most work in zebrafish has focused on the regulatory subunit of the myosin phosphatase called Mypt1. In this work, we examined the critical role of Protein Phosphatase 1, PP1, the catalytic subunit of the myosin phosphatase.

**Methodology/Principal Findings:**

We observed that in zebrafish two paralogous genes encoding PP1β, called *ppp1cba* and *ppp1cbb*, are both broadly expressed during early development. Furthermore, we found that both gene products interact with Mypt1 and assemble an active myosin phosphatase complex. In addition, expression of this complex results in dephosphorylation of the myosin regulatory light chain and large scale rearrangements of the actin cytoskeleton. Morpholino knock-down of *ppp1cba* and *ppp1cbb* results in severe defects in morphogenetic cell movements during gastrulation through loss of myosin phosphatase function.

**Conclusions/Significance:**

Our work demonstrates that zebrafish have two genes encoding PP1β, both of which can interact with Mypt1 and assemble an active myosin phosphatase. In addition, both genes are required for convergence and extension during gastrulation and correct dosage of the protein products is required.

## Introduction

Reversible phosphorylation of the type II myosin regulatory light chain (MLC2) is a critical regulatory mechanism for controlling type II myosin and the actin cytoskeleton [1,2]. Precise control of MLC2 phosphorylation is required for numerous cellular processes including morphogenetic cell movements during development, smooth muscle contraction and tumor cell invasion [1,2]. MLC2 is phosphorylated, primarily at serine 19 but also threonine 18, by a number of protein kinases, including Myosin Light Chain Kinase (MLCK), Rho-Associated Protein Kinase (ROCK) and Zipper-Interacting Protein Kinase (ZIPK) [3,4]. The dephosphorylation of both sites on MLC2 is mediated by a highly conserved Myosin Phosphatase (MP) complex consisting of a targeting subunit Mypt1, the catalytic subunit Protein Phosphatase 1 β (PP1β) and an associated 20 kD protein (m20) [5,6]. MLC2 kinases and phosphatases are in turn precisely regulated by reversible phosphorylation in response to a variety of signaling pathways [6]. Importantly, myosin phosphatase is regulated by the kinases ROCK and ZIPK, which can phosphorylate Mypt1 at the conserved threonines 696 and 850 (amino acid numbering in the human protein) inhibiting myosin phosphatase activity [7]. Myosin phosphatase is also inhibited by the CPI-17 family of small inhibitory proteins [8]. In addition, phosphorylation of Mypt1 is regulated by non-canonical Wnts and downstream RhoA/ROCK signaling [9].

Much of our understanding of myosin phosphatase regulation has stemmed from extensive studies in smooth muscle [3,10,11]. However, the role of myosin phosphatase regulation in non-muscle cells has been less well studied [5]. While genetic studies in invertebrates have greatly expanded our understanding of myosin phosphatase during development [12–15], many of the myosin phosphatase regulatory mechanisms are specific to vertebrates [5]. Genetic experiments in mice have proven difficult because both MYPT1 knockout mice [16] and mice with a retroviral insert in PP1β (gene trap OST319712, Lexicon Genetics, http://www.mmrrc.org) die early in embryonic development and have therefore proven difficult to characterize. A conditional knockout of Mypt1 in mice has recently been generated and used to analyze smooth muscle function, but the non-muscle developmental roles of Mypt1 remain a mystery [17]. The zebrafish has proven to be an ideal vertebrate genetic model organism to study non-muscle myosin phosphatase function particularly in early development [9,18–20]. Either mutation or morpholino anti-sense oligonucleotide knockdown of zygotic *mypt1* (*ppp1r12a*) expression causes failure of liver development, disorganized somites and over-contractility of the neural epithelium leading to neural fold defects [18,19]. Knockdown of both maternal and zygotic expression of *mypt1* using morpholino antisense oligonucleotides results in morphant embryos with hypercontracile mesodermal cells that fail to undergo proper morphogenetic cell movement during gastrulation [9,20]. The zebrafish mutant *sq181* is a *mypt1* loss-of-function allele containing an altered

PP1-binding motif, which is modified from KVKF to KMKF [18]. The K/R–V/I-X-F motif mediates the binding of several PP1 binding proteins to a hydrophobic pocket on the catalytic subunit of PP1 [21,22]. Also, splice-blocking morpholino knockdown of *ppp1cba* mimics the liverless phenotype of *mypt1* mutants [18], indicating that myosin phosphatase activity is dependent on both Mypt1 and PP1.

The earliest phenotype of Mypt1 morphants is a failure to undergo proper convergent extension (CE) during gastrulation [9]. CE is a major driving force in vertebrate gastrulation and is a mechanism by which cells first move towards the future dorsal side of the embryo and then intercalate between neighboring cells, resulting in an overall dorsal-ventral narrowing (convergence) and anterior–posterior lengthening (extension) of the embryonic tissue [23–28]. Precise regulation of cellular contractility is required for mesodermal cells to undergo proper CE movements and cell behavior changes, and gain- or loss-of-functions of members of the Rho-Rock-Mypt1-Myosin pathway result in severe gastrulation defects [9,29,30]. Disruption of Rho-Rock signaling or the upstream Wnt-PCP pathway results in a characteristic phenotype of a shortened and broadened body axis, reduced axial migration of the prechordal plate, disruption of cell polarity and changes in cellular protrusive activity [9,20,29,30].

PP1 is an essential eukaryotic protein phosphatase that regulates numerous physiological processes including, cellular contractility, cell cycle, gene expression, protein synthesis and neuronal signaling [21]. PP1 is regulated through specific interactions with a large number of regulatory and inhibitory subunits [31]. Many organisms have multiple genes for PP1; the protein products are referred to as PP1 isoforms. Interestingly, all three mammalian genes (PP1α, PP1β and PP1γ) appear to have nearly ubiquitous expression in all organisms examined, and their amino acid sequences have diverged very little, although a few important splice variants have been observed [32]. Interestingly, zebrafish have five PP1 genes: two genes similar to PP1α, two genes similar to PP1β and one gene related to PP1γ. Genetic experiments in flies and mice have indicated that PP1 isoforms have both unique isoform-specific functions and other roles that overlap with the other PP1 isoforms [33,34]. While all three mammalian PP1 isoforms can partially complement the single essential PP1 catalytic subunit in *S. cerevisiae*, the resulting yeast still display distinct phenotypes indicating partial PP1-loss-of-function even though they retain nearly identical in vitro phosphatase activity [35]. Throughout eukaryotic evolution it appears that regulatory and inhibitory subunits proliferate and diverge at a much higher rate than the catalytic subunits, again indicating that divergence in function of PP1 isoforms is a slow and difficult process [32]. Isoform specific regulation of PP1 is an active area of research and the regulatory mechanisms at play have been examined in only a handful of holoenzyme forms of PP1, such as the Mypt1-PP1β complex and the Neurabin-PP1γ complex [36–39]. In both cases the regulatory subunit preferentially interacts with one of the three PP1 genes, and this interaction is regulated by a short amino acid sequence adjacent to the PP1 binding site. How other holoenzymes interact with PP1 in an isoform specific manner remains to be addressed [31,40].

In this work, we set out to examine the essential catalytic subunit of the zebrafish myosin phosphatase. We observed that zebrafish, in contrast to mammals, which have a single PP1β gene, has two broadly expressed paralagous genes for PP1β, termed *ppp1cba* and *ppp1cbb*. Early in the evolution of teleosts, the genome underwent a duplication event which caused a large number of gene duplications [41]. Many of these redundant genes have been lost during evolution, while in other cases both genes have been retained, some remaining redundant while others diverge functionally [42]. Importantly, the zebrafish PP1β proteins, termed PP1Ba and PP1Bb, are highly homologous at the amino acid level, and all residues involved in Mypt1 binding and catalysis are conserved [36]. The effect of active myosin phosphatase on human cells has been well characterized [43,44], thus we can use cultured cells to test whether both genes encode for a protein that can assemble an active myosin phosphatase. In both GST pull down and immunoprecipitation experiments, the two isoforms of PP1β could interact with similar affinity to zebrafish Mypt1. In addition the assembled complex could induce the dephosphorylation of purified MLC2. Expression of both Mypt1 and either PP1 isoform in HeLa cells resulted in significant reorganization of the actin cytoskeleton and greatly reduced MLC2 phosphorylation. Knockdown of either *ppp1cba* or *ppp1cbb* individually or of both genes simultaneously resulted in severe defects in morphogenetic cell movements during gastrulation. Interestingly, co-injection of mRNA for either *ppp1cba* or *ppp1cbb* could rescue the gastrulation defect, indicating that these genes have the same function during early development but that gene dosage is critical for gastrulation. Furthermore, based on a strong genetic interaction with Mypt1 the gastrulation defect seen in *ppp1cba*/*ppp1cbb* knockdown embryos appears to be due to a failure to assemble an active myosin phosphatase complex. Based on these results we can conclude that both PP1Ba and PP1Bb form active myosin phosphatase complexes with Mypt1 and are critically required for proper cell movement during early development in vertebrates.

## Materials and Methods

### Zebrafish and ethics

Wild-type Zebrafish (*Danio rerio*) embryos were obtained through natural spawning and were maintained and staged as described previously [45]. All experiments were approved by and conducted in accordance with the guidelines established by the Institutional Animal Care and Use Committee at the University of the Pacific, IACUC approval number: 10R03.

### Cell Culture

HeLa and HEK293T cells (obtained from the ATCC) were maintained in Dulbecco’s Modified Eagle Medium (DMEM) containing 25 mM D-glucose and 1 mM sodium pyruvate supplemented with 10% Fetal Bovine Serum (Invitrogen - Gibco). All cells were maintained in a 5% CO_2_ incubator at 37^o^C. Transfections were performed using Lipofectamine LTX reagent (Invitrogen) using manufacturer’s instructions. Cells were plated in 6-well petri dishes on coverslips coated with 1 µg of fibronectin (Sigma F1141) and transfected with 0.5 µg DNA for all plasmids except ZIPK which was 0.25 µg.

### Plasmids and Cloning

Zebrafish *ppp1cba* (NP_001004527) and *ppp1cbb* (BC116539) were amplified from total cDNA of 24 hpf zebrafish embryos generated as described [46]. The cDNA for *mypt1/ppp1r12a* (NM_001003870) and the mutant cDNA for *sq181* were the kind gift of Jinrong Peng, Proteos, Singapore. The ORF for both *ppp1cba* and *ppp1cbb* were subcloned into expression vectors pEGFP-C1 and pCS2. The N-terminus (1-300 aa) of Mypt1 with the PP1β binding motif (KVKF) was subcloned into pCS3-MT (Myc-tag) vector and the pGEX 4T-1 protein expression vector (GE Healthcare). Two versions of MYPT1 with mutated PP1β binding motifs (KMKF and KAKA) were cloned into pCS3-MT (Myc-tag) vector and the KMKF version was cloned into pGEX4T-1. Mutant constructs were generated using a quikchange site-directed mutagenesis kit (Stratagene). GFP-tagged mammalian PP1β was kind gift of Mathieu Bollen and Janina Goernemann [47].

### Morpholinos

Morpholinos for *ppp1cba* and *ppp1cbb, p53* and the standard control morpholino were obtained from genetools and the Mypt1 morpholino was described previously [9]. The sequences are as follows: MO *ppp1cba* GTTTAATTCCCCCTCCGCCATCTTC, MO *ppp1cbb* ctccgccatgactgactgaccgact, control MO CCTCTTACCTCAGTTACAATTTATA, *p53* MO GCGCCATTGCTTTGCAAGAATTG, MO *mypt1* GGCGTCCGCCATCTTCATCCCCTCG. 5-base mismatch control morpholinos to *ppp1cba* and *ppp1cbb* were also designed with the following sequences: GTTTCCTTCCCCCTAAGCCATGTTC and CTCCGAAATGACTGGTTGACCCACT. Alternate translation blocking morpholinos were designed to *ppp1cba* and *ppp1cbb* with the sequences: ATCTTCAGTGCCGAGTTCTGCATGC and GACGGAGACCCGCCCTCACGCGCGC.

### In Situ hybridization

Whole-mount in situ hybridization was performed using digoxigenin-labeled antisense and sense RNA probes for *ppp1cba* and *ppp1cbb* generated from pCS2 vector clones. To avoid cross-reactivity, embryos and probes were processed under highly stringent conditions as described previously [9], with 0.05X SSC and 65^0^C stringency washes. Such a high stringency would require near identity for staining and based on standard Tm calculations [48] would be predicted to prevent cross reactivity between *ppp1cba* and *ppp1cbb* probes [49]. Probes for analysis of convergence and extension and dorsal ventral patterning were used as described in [29,45] and were used at standard stringency (0.2X SSC at 65^0^C).

### RT-PCR

RNA was isolated using the E.Z.N.A. ® Total RNA Kit I (Omega Bio-Tek). cDNA was prepared using 1 µg RNA using manufacture’s instructions. Transcript specific primers were used to amplify *ppp1cab* GTGCGAGGATGTCGTCCTGGGAAG (sense); TCTTCTTGGGGGCTTGGGC (antisense), *ppp1caa* ATGGCGGAGGGCGAGCTGG (sense); GCTTCTTTGGGGGCGTGG (antisense) and *ef1a* TCACCCTGGGAGTGAAACAGC (sense); ACTTGCAGGCGATGTCAGCAG (antisense). For all three, 22 PCR cycles were performed.

### Protein Purification

GST expression constructs for WT and mutant Mypt1 or GST-MLC2 (a kind gift of Dr. Ruey-Hwa Chen at the Institute of Biological Chemistry, Academia Sinica, Taipei, Taiwan) were transformed into *E coli* BL21 (DE3) pLysS (Invitrogen). Single colonies of transformed *E. coli* were used to inoculate 50 ml overnight cultures in LB-Miller broth with 100 µg/ml ampicillin and 33 µg/ml chloramphenicol in a shaking incubator at 37^o^C. The overnight culture was then used to inoculate 1 liter of broth and GST-Mypt transformed *E. coli* were grown at 30^o^C and GST-MLC2 at 37^o^C until an O.D. 600 of 0.5 was reached. The cultures were then induced with 0.1 mM isopropyl 1-thio-β-d-galactopyranoside (IPTG) for 3 hours. The cells were sedimented by centrifugation at 3000 × g for 10 min and resuspended in 10 ml of ice-cold buffer containing 10 mM Tris-HCl (pH 7.5), 150 mM NaCl, 1 mM EDTA, 0.1% (v/v) β-mercaptoethanol, 1 mg/ml lysozyme, 0.1% (w/v) Triton X-100 plus protease inhibitors (Fisher 78410). The resuspended cultures were lysed by sonication, and cell debris was removed by centrifugation at 20,000 × g for 10 min. Bacterial lysates were incubated with glutathione-Sepharose (Amersham Biosciences) for 1 h at 4°C. Beads were rinsed three times with 50 ml each of lysis buffer, followed by 10 mM Tris-HCl (pH 7.5), 150 mM NaCl, 0.1% (v/v) β-mercaptoethanol, 1 mM EDTA, and 1 mM EGTA. GST fusion proteins were eluted with 100 mM Tris-HCl (pH 8.0), 150 mM NaCl, and 10 mM glutathione. Eluted proteins were dialyzed into 50 mM Tris-HCl (pH 7.5), 0.1% (v/v) β-mercaptoethanol. Protein concentration was determined using Biorad, Bradford assay reagent following the manufactures instructions using bovine serum albumin as a control.

### GST Co-sedimentation

Equal concentrations of GST alone, GST fused wild type Mypt1 1-300 (KVKF) and Mypt1 1-300 mutant (KMKF) proteins were incubated with lysed HEK293T cell lysates expressing either GFP, PP1Ba or PP1Bb proteins in NETN buffer (20 mM Tris-HCl pH 8.0, 100 mM NaCl, 1 mM EDTA, 0.5% NP-40 plus protease inhibitors). Upon addition of glutathione beads (GTH) the pellet was washed with GST Wash buffer (50 mM Tris-HCl pH 7.5, 150 mM NaCl, 1 mM EDTA, 0.1% 2-mercaptoethanol and 0.1% NP40) and proteins were eluted at 95°C for 5 minutes in SDS-PAGE loading dye. All pulldowns were repeated at least 3 times with a representative western blot displayed in the figure.

### Co-Immunoprecipitation

15 hours post transfection cells were washed in PBS and lysed in NETN buffer. 3 µL of anti-myc (Covance, MMS-150R) or anti-GFP (Santa Cruz, B-2) antibody was used for immunoprecipitations to either pull down with MYPT1 or PP1β. 10 µL Protein G sepharose beads (GE Healthcare) were mixed with lysate and allowed to incubate at 4°C. Alternatively, protein complexes were immunoprecipitated using anti-myc agarose coupled beads to remove background immunoglobulins (Millipore). Proteins bound on the beads were washed with NETN and then eluted by addition of SDS-PAGE loading dye and heated at 95°C for 5 minutes. Proteins were separated by 12% SDS-PAGE and blotted with either anti-myc or anti-GFP antibodies (1:1000 dilutions). All co-IPs were repeated at least 3 times with a representative western blot displayed in the figure.

### Phos-tag-gel electrophoresis

Phos-tag acrylamide (Wako Pure Chemical Industries) was used for SDS, Page to determine the extent of myosin light chain phosphorylation as described [50] and detected with either an anti MLC2 antibody (Cell Signaling #3672) or coomassie staining (Bio-Safe^TM^ Biorad). Purified GST-MLC2 was phosphorylated for 30 minutes using flag-tagged zebrafish ZIPK (a kind gift of Adi Kimchi, Weizmann Institute of Science, Rehovot, Israel) immunoprecipitated from HEK293T cells [51]. Each phosphorylation was performed in 50 µl kinase buffer (25 mM Tris-HCl pH 7.5, 5 mM β-glycerophosphate, 0.1% 2-mercaptoethanol, 0.1 mM Na _3_VO_4_, 10 mM MgCl_2_ and 200 µM ATP) and 1 µg of purified GST-MLC2 bound to GTH beads. The phosphorylated MLC2 beads were then washed and used in phosphatase assays with either Mypt1 and PP1Ba or Mypt1 and PP1Bb immunoprecipitated from HEK293T cells. Dephosphorylation was performed for 30 minutes in 25 µl of phosphatase buffer (50 mM Tris-HCl pH 7.5, 0.1% 2-mercaptoethanol, 1mM EDTA).

### Stress Fiber Assay

HeLa cells were trypsinized and plated on fibronectin coated coverslips followed by transfections. Approximately 15-18 hours post transfections cells were fixed in 4% PFA, permeabilized in 0.2% Triton-X and washed with PBS. Cells were stained with Alexa 568 Phalloidin (Invitrogen) and DAPI (Molecular Probes). Assaying for stress fibers was performed essentially as described [29]. Briefly, a normal stress fiber phenotype was defined as cells containing multiple stress fibers passing through the majority of the cytoplasm. The moderate phenotype of reduced stress fibers was scored when cell displayed roughly normal cell size and shape but stress fibers were clearly absent from a majority of the cytoplasm but may remain along the periphery. A severe phenotype was scored when stress fibers were largely absent from the cytoplasm and periphery and large scale changes in cell shape and reductions in the cytoskeleton were observed.

### Immunofluorescence

HeLa cells were plated, fixed, permeabilized and washed as mentioned above. Cells were blocked with 2% BSA for 30 minutes at room temperature. Phospho-myosin light chain 2 antibody (Cell signaling, #3675) diluted (1:50) in 2% BSA was added and cells were incubated overnight at 4°C and washed three times with 1X PBS before addition of Alexa Fluor 568 anti-mouse secondary antibody (Invitrogen) diluted (1:1000) in 2% BSA. Cells were incubated for 1 hour at room temperature and washed three times with PBS followed by mounting and visualization by confocal microscopy.

### Microscopy

Slides from stress fiber assay, immunofluorescence assays and zebrafish DIC images were visualized using Leica DMIRE2 inverted fluorescence microscope using metamorph software, a Plan Apo 40X/0.85NA objective and Yokogawa CSU-X1 spinning disc confocal with a QuantEM: 5125C camera. A Leica S6D dissecting microscope with a DFC290 camera was used for in situ hybridization and zebrafish phenotype analysis and images were acquired using Leica application suite.

### Zebrafish injection and analysis of embryo phenotype analysis

Zebrafish embryos were injected with the indicated concentration of morpholino or mRNA at the one-cell stage as described [45] with needles calibrated to inject 1 nl of fluid. A detailed description of zebrafish embryo care and analysis of morphogenetic cell movement is available here [45]. Briefly, embryos were kept in E3 media at 28.5^o^C until the desired stage. To determine defects in body axis elongation, 48hr embryos were analyzed using a dissecting microscope and camera. The anterior–posterior length of the embryo was determined using the distance measure tool on ImageJ. The length of the experimental embryos was then normalized to uninjected embryos from the same clutch and the data was reported as % of control. For measurement of convergent extension, bud stage embryos were mounted with a lateral view of the embryo. The angle between the leading edge of the polster and the end of the notochord was calculated using ImageJ.

### Zebrafish Embryo Lysis

25 zebrafish embryos were dechorionated using pronase and subjected to deyolking in (100 µl of 55 mM NaCl, 1.8 mM KCl, 1.25 mM NaHCO_3_) and pipetting until the embryos are disrupted. The lysate was then centrifuged at 300 g for 1 minute to precipitate the unlysed cells and separate them from the excessive yolk proteins. The embryos were then lysed in 1X SDS-PAGE loading buffer and subjected to western blot analysis using anti-PP1β (Millipore) and anti-α-tubulin (Cell Signaling #3873) antibodies.

### Mesodermal cell behavior

A detailed protocol for imaging zebrafish mesodermal cells is available elsewhere [45]. Briefly, embryos were mounted in methylcellulose at the indicated stage and observed with a 40x objective using DIC microscopy. Length-width ratios were calculated using Photoshop by measuring the dorsal-ventral length of either axial or presomitic mesoderm and dividing by the anterior–posterior width. Three to five randomly selected morpholino-injected and rescued embryos were chosen and 25 cells were measured from each embryo and compared to uninjected clutch-mates.

### Homology and Phylogenetic analysis

The sequences of various PP1β proteins were aligned using ClustalX and boxshade (http://www.clustal.org) (http://www.ch.embnet.org/software/BOX_form.html). The phylogenetic tree was generated using the maximum likelihood method in the PhyML program (http://www.phylogeny.fr) using settings as described previously [52].

The accession numbers for the proteins used are as follows: *D. melanogaster* Flapwing NP_524738, *H. sapiens* PP1B NP_002700, *D. rerio* PP1Ba NP_001004527, *D. rerio* PP1Bb BC116539, *S. salar* PP1Bb NP_001135160, *S. salar* PP1Ba ACN58678, *M. musculus* PP1B NP_766295, *X* *. tropicalis* PP1B NP_001011467.

### Statistical Analysis

Statistical analysis was performed using MS Excel and Daniel’s XL Tool box (http://xltoolbox.sourceforge.net/. For quantitative data, a mean and standard error are shown and statistical significance was determined using a 1 factor ANOVA and Tukey post hoc comparisons.

## Results

### PP1β genes are ubiquitously expressed during early zebrafish development

**Figure 1:**
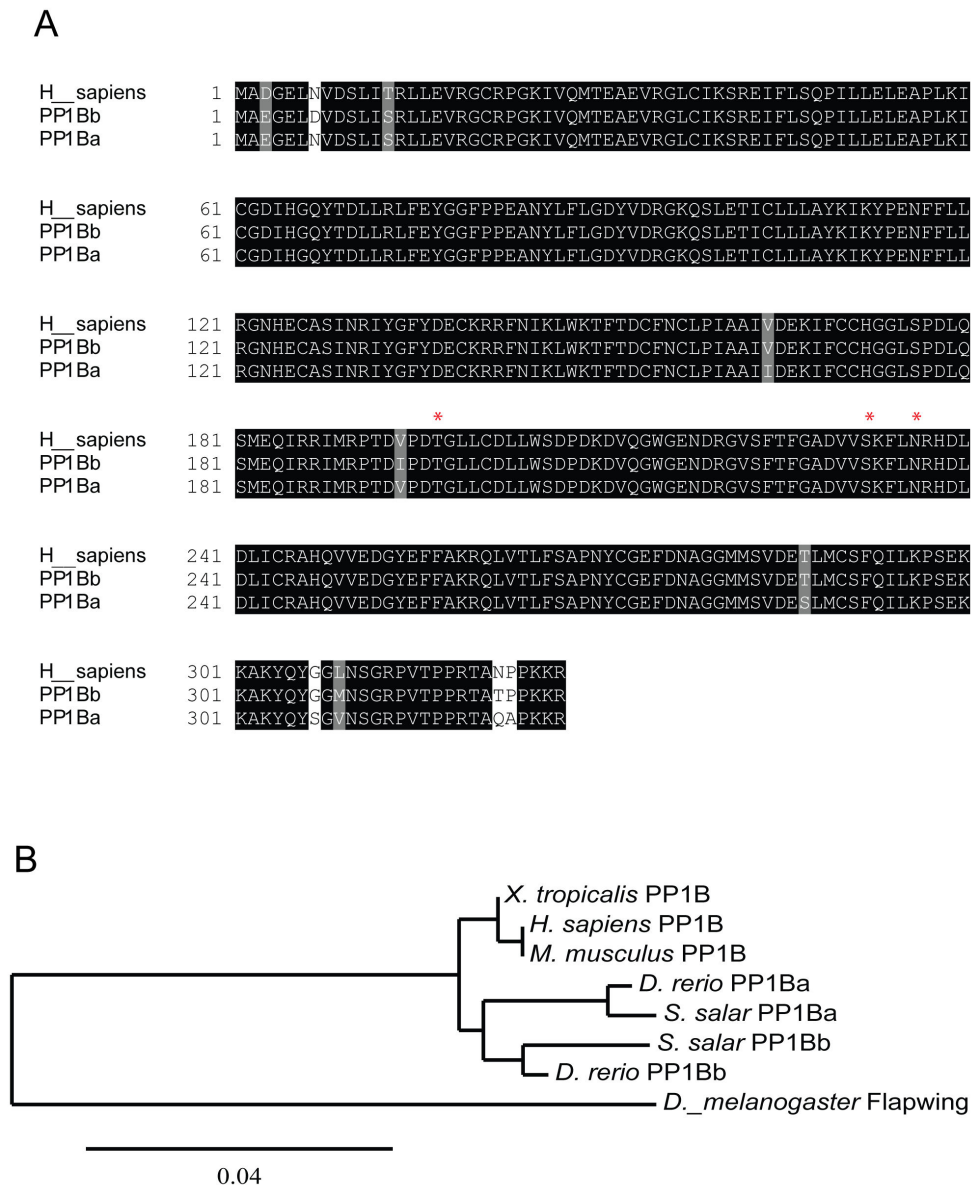
Zebrafish have two paralogs for Protein Phosphatase 1 β. A. A protein sequence alignment of zebrafish PP1Ba and PP1Bb with human PP1β. The amino acids marked with a * are critical for isoform specific interaction with Mypt1. B. Phylogenetic analysis of vertebrate PP1β genes using the maximum likelihood method with *Drosophila* Flapwing (the Drosophila PP1β) as an outgroup.

In order to better understand the regulation and function of the myosin phosphatase in early zebrafish development we cloned the cDNAs for zebrafish *ppp1cba* and *ppp1cbb* from 24 hpf zebrafish embryos. An amino acid sequence alignment of PP1Ba and PP1Bb demonstrates a high degree of conservation with only 8 amino acids divergent between the two proteins (Figure 1A). Importantly, the core catalytic domain is especially conserved with only two conservative substitutions. All other amino acid differences are in the N- or C-terminal tails, however, the amino acids Threonine 197, Serine 232 and Asparagine 236, which are critical for the β-isoform specific interaction with Mypt1 [36], are all conserved. Phylogenetic analysis of PP1β genes from a variety of animals is consistent with a proposed duplication in the fish linage as indicated by the presence of *ppp1cba* and *ppp1cbb* in both zebrafish and salmon (Figure 1B).

**Figure 2:**
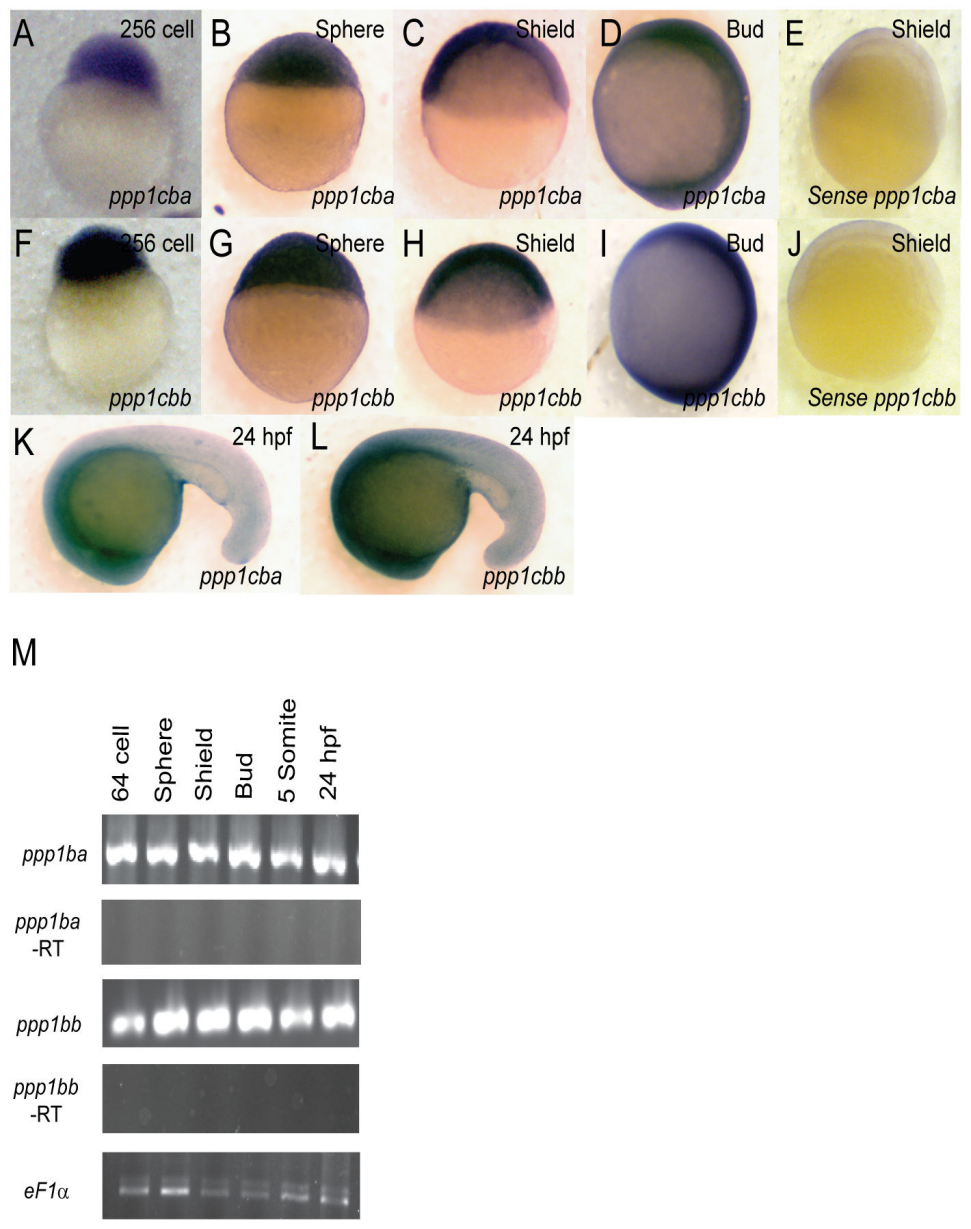
Embryonic Expression of *ppp1cba* and *ppp1cbb* during zebrafish development. Detection of *ppp1cba* and *ppp1cbb* mRNA was carried out by whole-mount in situ hybridization using gene-specific probes on staged embryos from 256 cells to 24 hpf. Images are lateral views, animal pole at top. (A–L) *ppp1cb* transcripts are ubiquitously expressed at early developmental stages, 256 cells stage (A and F), sphere stage (B and G), shield stage (C and H), bud stage (D and I) and 24 hpf (K and L). A probe for *ppp1cba* was used in (A–D and K) while *ppp1cbb* was used for (F–I and L). Negative control sense probes for *ppp1cba* and *ppp1cbb* did not show staining (E and J). Gene specific primers were used to detect *ppp1cba* and *ppp1cbb* in various stages of development by RT-PCR. (M) Both genes were expressed maternally and zygotically throughout early development. Amplification of *eF1α* and total RNA without addition of reverse transcriptase were used as controls.

In order to determine the spatiotemporal expression of PP1β isoforms, the cDNA for both zebrafish isoforms were used to produce probes for in situ hybridization. The genes are highly similar in sequence (83% in the ORF). Thus all hybridizations were performed at a high stringency that would be predicted to prevent cross reactivity of the probes [48,49]. Both *ppp1cba* and *ppp1cbb* were expressed maternally and zygotically (Figure 2 A–L) and were ubiquitously expressed during the 256 cell stage (Figure 2 A and F), sphere stage (Figure 2 B and G), shield stage (Figure 2 C and H), bud stage (Figure 2 D and I) and 24 hpf (Figure 2 K and L). At 24 hpf both isoforms are enriched in the head regions, with lower level expression in the tail (Figure 2 K and L). To control for background staining, sense probe was tested on each stage and developed for the same length of time as the anti-sense probe. No significant background staining was observed at shield stage (Figure 2 E and J) or other early stages (data not shown). While the in situ hybridization was performed at the highest stringency we cannot completely rule out some cross-reactivity to the probes. Thus, we confirmed the maternal and zygotic expression with RT-PCR using gene specific primers (Figure 2 M). Importantly, primers designed to each gene did not cross-react with the others when tested against purified cDNA (data not shown). Interestingly, this expression pattern for both *ppp1cba* and *ppp1cbb* closely matches the observed expression pattern for *mypt1* [18].

### Zebrafish PP1Ba and PP1Bb both interact with Mypt1

**Figure 3:**
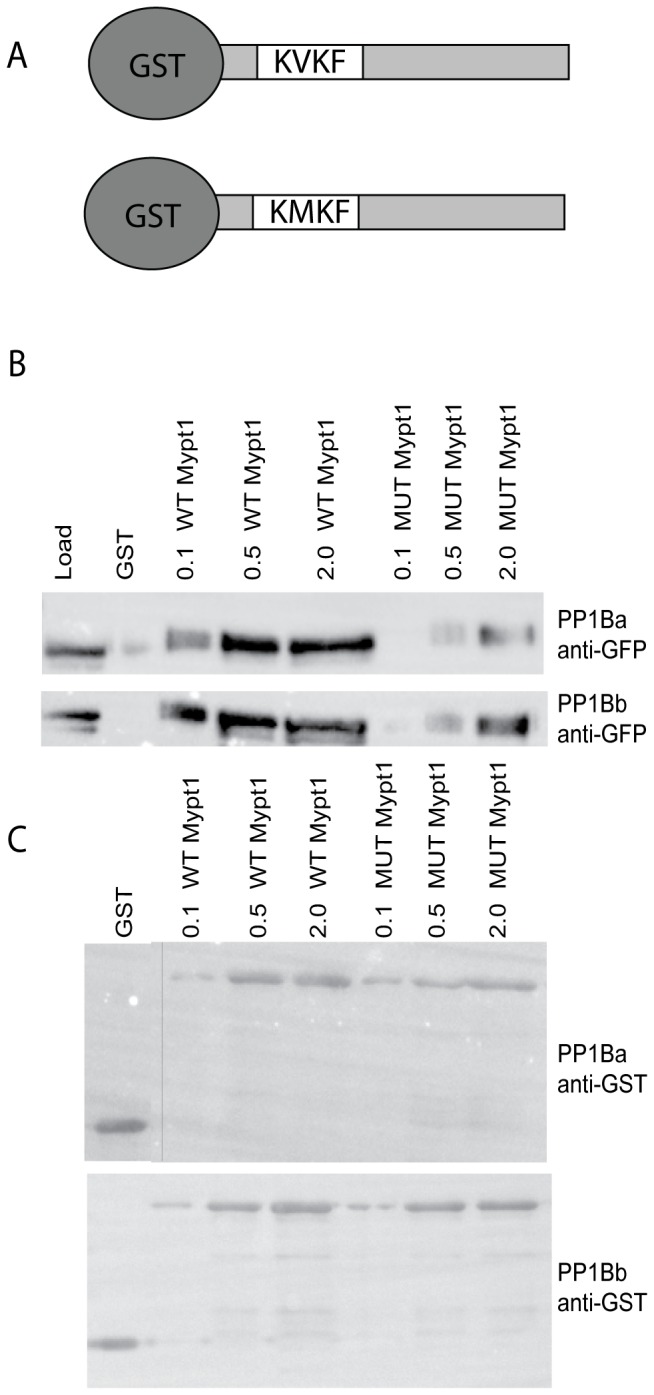
PP1Ba and PP1Bb interact with Mypt1 in vitro. (A) Schematic of GST fusion proteins used for co-sedimentation. The N-terminal 300 amino-acids of zebrafish Mypt1 are fused to GST with either a WT KVKF or mutated KMKF PP1 binding motif. (B and C) Increasing concentrations of purified GST fusion proteins (indicated protein added in micrograms) were incubated with glutathione sepharose and used to sediment GFP-tagged PP1Ba and PP1Bb from HEK293T cell lystates. GST alone was used as a negative control. The co-sedimented proteins were analyzed by SDS-PAGE and immunoblotted using an anti-GFP (B) or anti-GST antibody (C). A vertical bar indicates the presence of a lane removed from the western blot in editing.

We next set out to determine if these PP1β isoforms could functionally interact with Mypt1. To test this we used two complementary approaches, GST pulldowns and co-immunoprecipitations (co-IPs). In order to carry out GST pulldowns, recombinant N-terminal fragments of zebrafish Mypt1 were expressed and purified from *E. coli* as GST fusion proteins. Both a WT and the *sq181* allele of Mypt1 with Valine 36 mutated to Methionine (KMKF), which mutates the PP1 binding domain, were purified. These purified proteins were then used to sediment zebrafish GFP-PP1Ba and GFP-PP1Bb expressed in HEK293T cell lysates. We observed that both PP1Ba and PP1Bb interacted with WT Mypt1 with similar affinity but showed reduced affinity for the mutant Mypt1 (Figure 3). No interaction was seen between GST alone and PP1β isoforms (Figure 3) or between GST-Mypt1 and GFP (data not shown)

**Figure 4:**
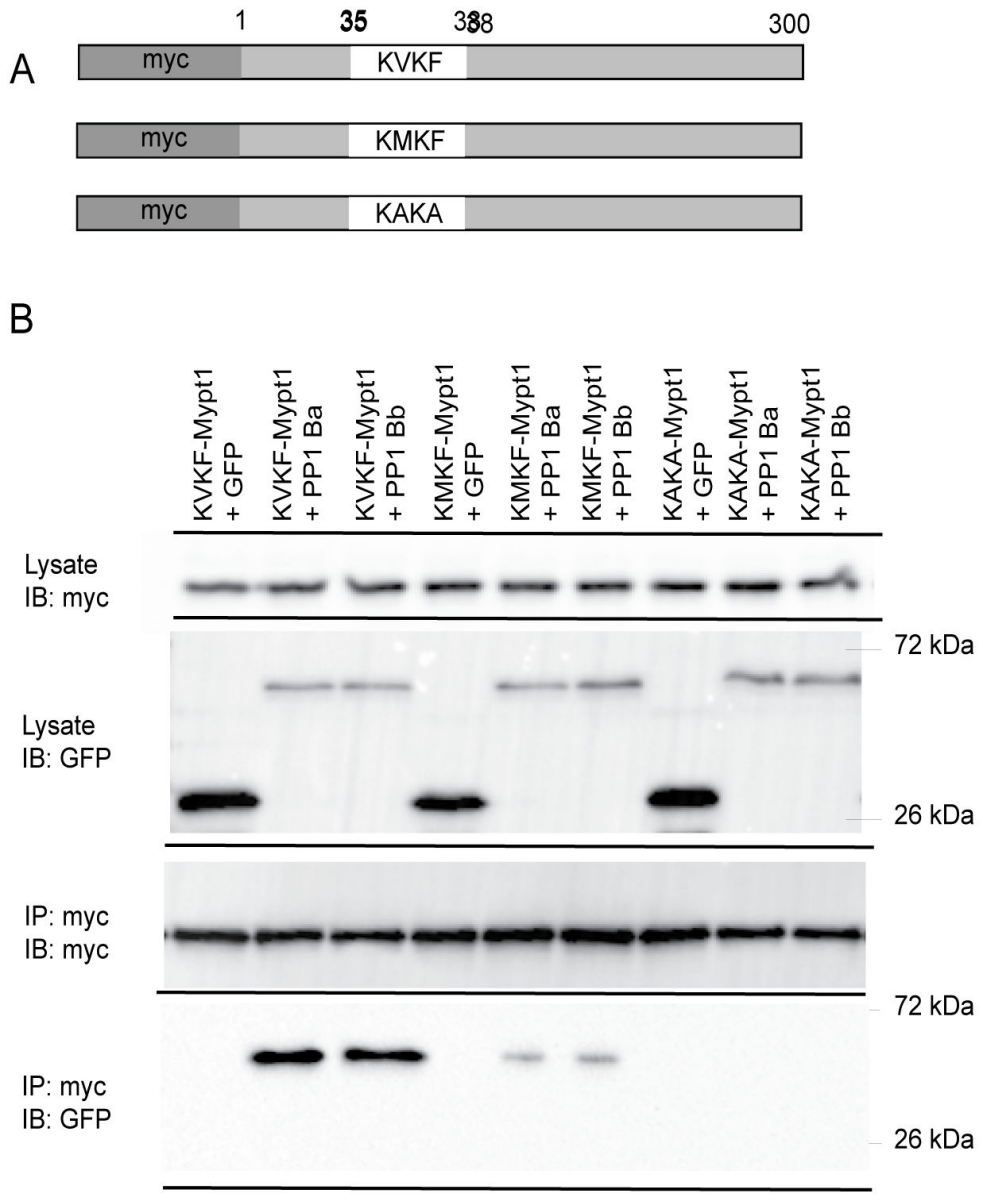
Interaction of Mypt1 and PP1β isoforms. (A) A schematic of Mypt1 constructs used in immunoprecipitation experiments. The sequence of the PP1 binding domain is indicated as the WT KVKF, the partial-loss-of-function KMKF and the null KAKA. (B) Myc-tagged Mypt1 and GFP-tagged PP1β proteins were expressed in HEK293T cells and immunoprecipitated with anti-myc antibodies. The IPs were immunoblotted with anti-GFP antibodies and anti-myc antibodies.

The *sq181* allele of *mypt1* (KMKF) results in a loss-of-function allele in zebrafish [18]; however, in our results residual binding affinity remained. We hypothesized that this was due to residual activity of the KVKF motif, a conserved PP1 binding domain found in Mypt1 and many other PP1 binding proteins [21,22,31]. In order to test this hypothesis, we generated a null Mypt1 mutant where Valine 36 and Phenylalanine 38 were both mutated to alanine, which we predicted would completely abolish PP1 binding. Myc-tagged versions of Mypt1 were expressed in HEK293T cells with either a WT KVKF motif or mutant KMKF or KAKA (Figure 4 A) and used in co-IP experiments with GFP-PP1Ba and GFP-PP1Bb or GFP alone. The IP was carried out with a myc antibody, and both PP1Ba and PP1Bb interacted with WT Mypt1 with similar affinities (Figure 4 B). Reduced binding was seen with the KMKF mutant, and no interaction of either isoform was seen with the KAKA mutant (Figure 4 B). No background interaction was seen with any mutant and GFP alone (Figure 4 B), and these interactions were confirmed with the reverse experiments using the anti-GFP antibody to IP and blotting with anti-myc (data not shown). These experiments clearly indicate that both PP1Ba and PP1Bb can interact with Mypt1. In addition, these results confirm that the *sq181* allele of Mypt1 is hypomorphic, exhibiting a partial-loss-of-function retaining some PP1 binding affinity.

### Expression of active myosin phosphatase results in rearrangement of the actin cytoskeleton

**Figure 5:**
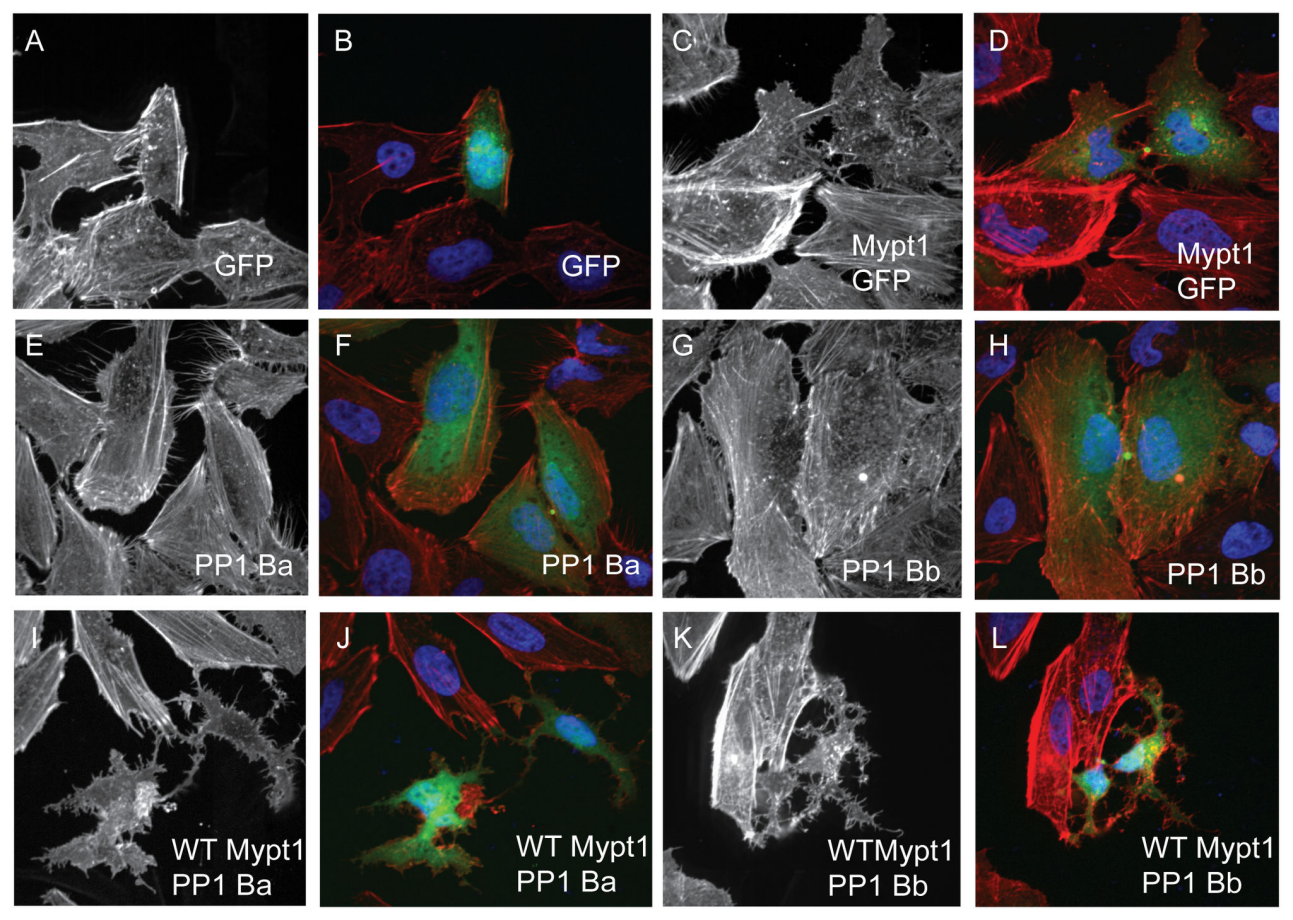
PP1Ba and PP1Bb assemble myosin phosphatase complexes that regulate the actin cytoskeleton. HeLa cells were transfected with either GFP alone (A, B), zebrafish Mypt1 (1-300) and GFP (C, D), zebrafish GFP-PP1Ba (E, F), zebrafish GFP-PP1Bb (G,H), Wild-type Mypt1 and PP1Ba (I, J), wild-type Mypt1 and PP1Bb (K, L). All cells were fixed and stained with DAPI and Alexa 568-phalloidin and imaged with confocal microscopy. Black and white images show phalloidin staining, while color images are a merge of DAPI (blue), GFP (green) and phalloidin (red).

Given that both isoforms of PP1β are capable of binding to Mypt1 with similar affinities, we set out to determine if the assembled complex is indeed an active myosin phosphatase. To test this we expressed myc-tagged zebrafish Mypt1 and/or GFP-tagged zebrafish PP1 constructs in HeLa cells. These cells were then stained with phalloidin and DAPI and imaged using confocal microscopy to visualize the actin cytoskeleton and nucleus, respectively. Thus we can directly assay the extent of myosin phosphatase activity of each PP1β isoform. Over-expression of an active mammalian myosin phosphatase caused a dramatic loss of stress fibers, a significant decrease in actin polymerization and an unusual pattern of retraction of the cell causing a neuronal-like phenotype [43]. Expression of GFP (Figure 5 A and B) resulted in no significant changes in the actin cytoskeleton relative to untransfected controls. Expression of Mypt1 (1-300) resulted in a modest reduction of stress fibers in transfected cells, but cell shape remained largely unchanged (Figure 5 C and D) (88% had reduced stress fibers, 0% had normal stress fibers and 12% had a severe cell shape change, n=25) presumably through interaction with endogenous HeLa cell PP1β. Either PP1Ba (Figure 5 E and F) (84% had reduced stress fibers, 8% had normal stress fibers and 8% had a severe cell shape change, n=25) or PP1Bb (Figure 5 G and H) (92% had reduced stress fibers, 4% had normal stress fibers and 4% had a severe cell shape change, n=25) alone result in more subtle changes in the actin cytoskeleton than Mypt1, with many of the cells still showing residual stress fiber formation mostly localized near the cell periphery. Co-expression of both Mypt1 and either catalytic subunit resulted in a dramatic loss of the normal stellate morphology of HeLa cells, and the cells contracted leaving a neuronal-like cell shape (Figure 5 I–L). WT-Mypt1 and either PP1Ba (72% had dramatically altered neuronal-like morphology with few or no stress fibers, 28% had reduced stress fibers but normal morphology and 0% had normal cell morphology, n=25) or PP1Bb (76% had dramatically altered neuronal-like morphology with few or no stress fibers, 24% had reduced stress fibers but normal morphology and 0% had normal cell morphology, n=25) resulted in a near complete loss of stress fibers and a dramatic reduction in overall actin polymerization. Additional controls were performed to confirm the functional interaction of Mypt1 and PP1β isoforms (Figure S1). Expression of the non-PP1 binding KAKA mutant of Mypt1 or the reduced KMKF mutant of Mypt1 did not results in large scale changes in stress fibers or cell shape (Figure S1). Expression of the KAKA mutant alone with either PP1Ba or PP1Bb resulted in a phenotype no more severe than the catalytic subunits alone (Figure S1). However, expression of either catalytic subunit with the reduced affinity Mypt1 mutant KMKF resulted in a phenotype more severe than either alone but less severe than with the WT Mypt1 (Figure S1), confirming residual myosin phosphatase function. Taken together, these data strongly indicate that expression of Mypt1 and either PP1Ba or PP1Bb results in an active complex that can dramatically alter the actin cytoskeleton, confirming that these proteins not only bind each other, but also form an active myosin phosphatases. These data also further demonstrate that the KMKF mutant of Mypt1 is a partial-loss-of-function with some residual activity, while the KAKA mutant is complete loss-of-function.

### Both PP1Ba and PP1Bb assemble active myosin phosphatase complexes that induce MLC2 dephosphorylation

**Figure 6:**
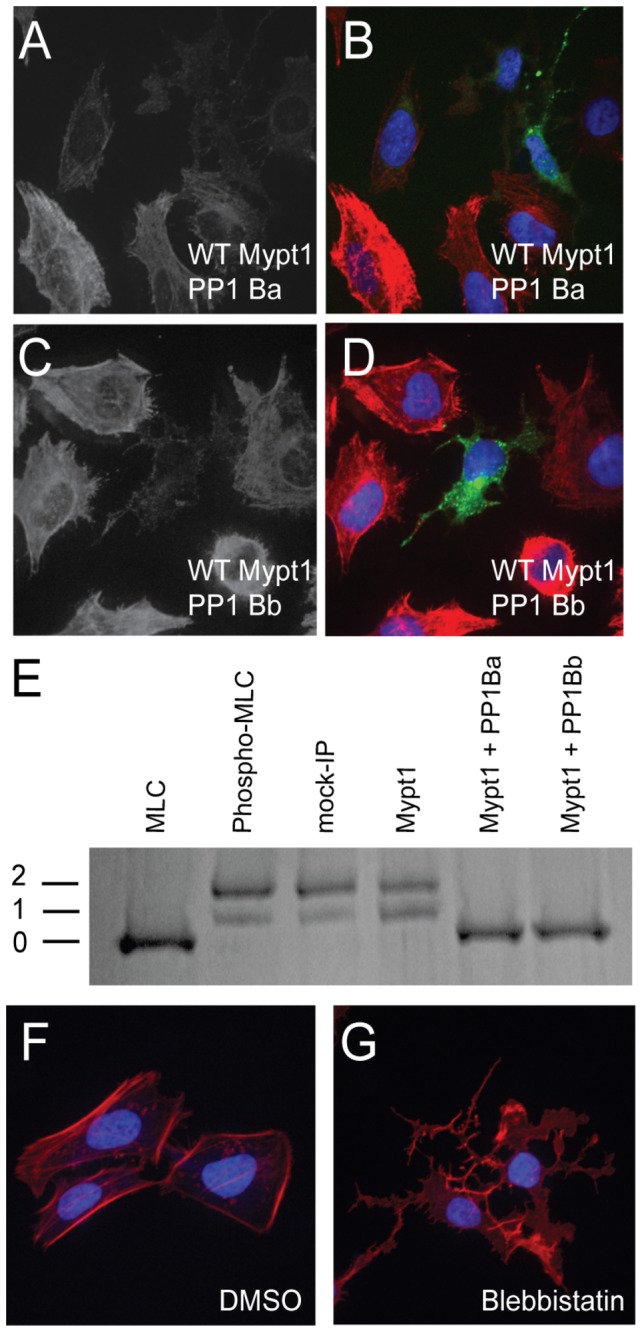
PP1Ba and PP1Bb dephosphorylate the regulatory myosin light chain. HeLa cells were transfected with zebrafish Mypt1 and GFP-PP1Ba (A, B) or zebrafish Mypt1 and GFP-PP1Bb (C, D). The HeLa cells were immunostained using an anti-phospho myosin light chain 2 antibody, co-stained with DAPI and imaged using confocal microscopy. Panels A and C show anti-phospho MLC2 staining, while B and D show a merge of GFP (green), phospho-MLC2 (red) and DAPI (blue). (E) Purified GST-MLC2 was run either as an untreated control (MLC2) or phosphorylated using ZIPK (Phosph-MLC2). The prephosphorylated MLC2 was dephosphorylated by Mypt1 (1-300)-PP1Ba or PP1Bb complexes immunoprecipitated from HEK293T cell lysates or using controls of treatment with beads from a myc-IP from untransfected cells (myc IP) or cell expressing only Mypt1 (1-300) and no additional catalytic subunit (Mypt1). Dephosphorylation (0), mono (1) and di-phosphorylation (2) was detected by band shift using a phos-tag SDS-PAGE gel as described in the materials and methods. HeLa cells were grown on fibronectin coated coverslips and treated with media containing either 0.1% DMSO (F) or 50 µM blebbistatin (G) for 4 hours. After treatment the HeLa cells were stained with DAPI (blue) and Alexa 568-phalloidin (red) and imaged with confocal microscopy and a color merged image is shown.

We next sought to demonstrate that the cell shape changes observed in HeLa cells were due to dephosphorylation of MLC2, confirming at a biochemical level that indeed these proteins form active holoenzymes. To test this we again expressed myc-tagged Mypt1 and GFP-tagged PP1 constructs in HeLa cells. These cells were then immunostained using an anti phospho-MLC2 antibody and co-stained with DAPI. Untransfected cells had strong phospho-MLC2 staining on the cell cortex and along stress fibers, while cells expressing Mypt1 with either PP1Ba or PP1Bb had reduced phospho-MLC2 staining (Figure 6 A–D). In addition, we sought to determine if the myosin phosphatase complexes could directly dephosphorylate MLC2 in vitro. To accomplish this GST-MLC2 was expressed in *E. coli* and purified. The purified protein was phosphorylated in vitro using flag-tagged Zip kinase immunoprecipitated from HEK293T cells. The phosphorylated GST-MLC2 was then incubated with myosin phosphatase complexes consisting of either Mypt1 alone, a mock purification from untransfected cells, Mypt1 and PP1Ba or Mypt1 and PP1Bb immunoprecipitated from HEK293T cells. After incubation with the phosphatase, the MLC2 protein was subjected to phos-tag SDS PAGE and stained with Coomassie (Figure 6 E) or visualized by western blot using an anti-MLC2 antibody (data not shown). The phos-tag gel allows for greater band shift of the phosphorylated MLC2 and mono, di and unphosphoryated MLC2 can be resolved [50]. Both PP1Ba and PP1Bb complexes resulted in rapid dephosphorylation of MLC2 with diphosphorylated MLC2 disappearing within 10 minutes (data not shown) and all bands collapsing to unphosphorylated after 30 minutes (Figure 6 E). Mypt1 alone did not result in the collapse of phosphorylation in 30 minutes (Figure 6E), but did result in complete dephosphorylation at longer incubation times (data not shown). This dephosphorylation was likely caused by Mypt1 binding to endogenous PP1β. Finally, treatment of HeLa cells with 50 µM of the non-muscle myosin II inhibitor blebbistatin caused cells to take on the collapsed neuronal-like morphology seen in cells overexpressing Mypt1 and PP1β isoforms indicating that the collapsed cell morphology is indeed caused by loss of type-II myosin function (Figure 6 F and G). Taken together these data provide strong evidence that both PP1Ba and PP1Bb can form active myosin phosphatase complexes with Mypt1 and dephosphorylate the myosin regulatory light chain.

### Zebrafish ppp1cba and ppp1cbb genes are required for body axis elongation during early zebrafish development

**Figure 7:**
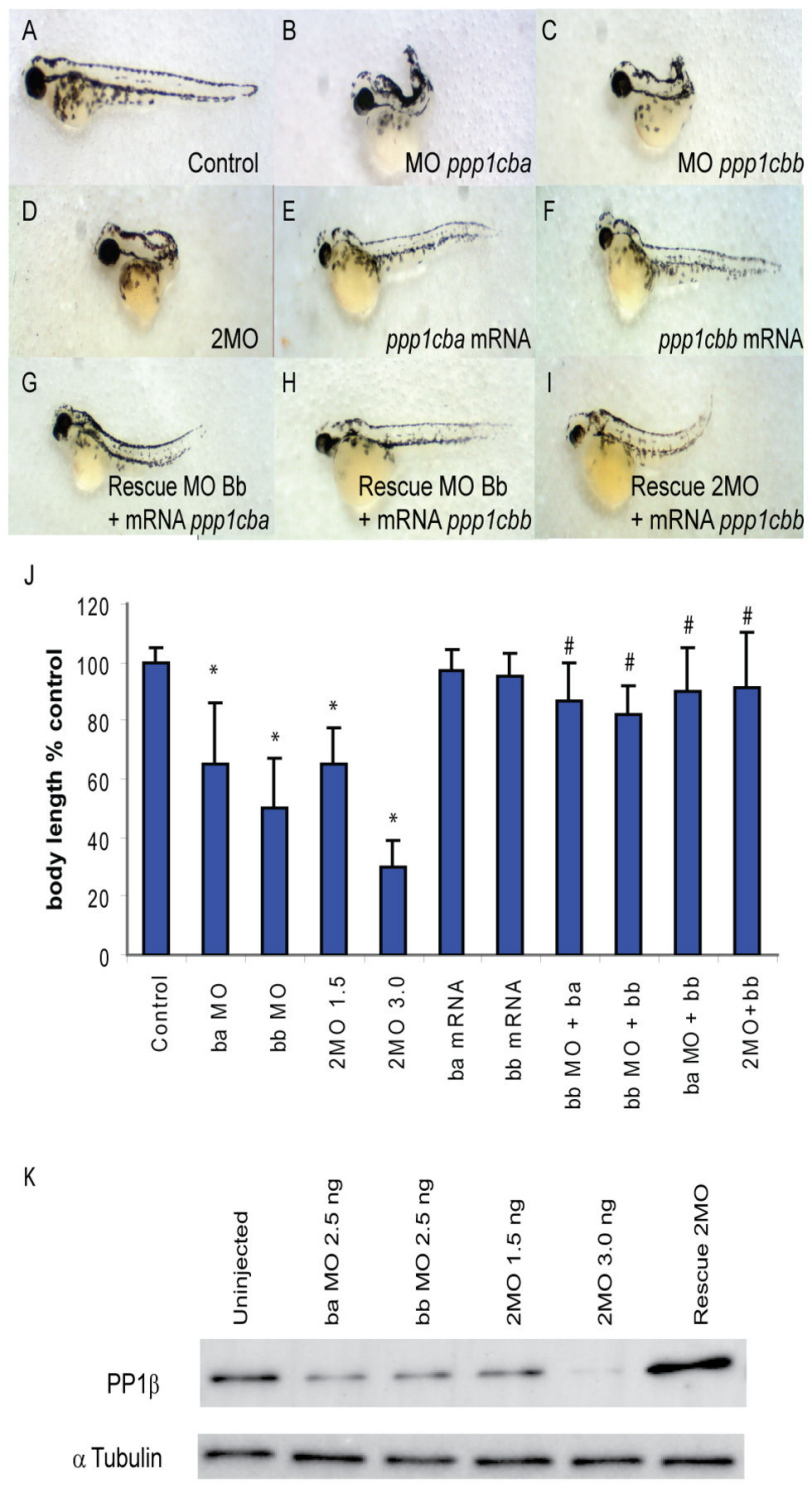
PP1β paralogs are required for proper zebrafish body axis elongation. (A–I) Lateral views of representative 48 hpf zebrafish embryos injected with (A) uninjected control, (B) 2.5 ng *ppp1cba* MO, (C) 2.5 ng *ppp1cbb* MO, (D) a mixture of 0.75 ng *ppp1cba* MO and 0.75 ng of *ppp1cbb* MO (2MO), (E) 200 pg of *ppp1cba* mRNA, (F) 200 pg of *ppp1cbb* mRNA (G) a partially rescued embryo injected with 100 pg *ppp1cba* mRNA and 2.5 ng of *ppp1cbb* MO (H) a partially rescued embryo injected with 100 pg *ppp1cbb* mRNA and 2.5 ng of *ppp1cbb* MO (I) a partially rescued embryo injected with 100 pg *ppp1cbb* mRNA and 0.75 ng of *ppp1cbb* and 0.75 *ppp1cba* MO (J) Quantification of the truncated body axis phenotype in morphant and mRNA injected embryos. Each injection was performed multiple times with 50 embryos used to calculate body axis length and reported as % of uninjected clutch mates. Error bars are standard error and a black * indicates a statistically significant difference from control and a # indicates a statistically significant rescue compared to the corresponding morpholino injected embryos. Statistical significance was calculated using a one-factor ANOVA with Tukey post hoc analysis and is defined as p < 0.05. (K) A western blot showing PP1β levels in zebrafish embryo lysates from control and embryos injected with two doses of morpholino cocktail (1.5 ng and 3.0 ng of total MO), individual *ppp1cba* or *ppp1cbb* (2.5 ng of either) morpholinos or a rescued embryo injected with 0.75 ng of each morpholino (1.5 ng total) and 100 pg of *ppp1cbb* mRNA. Tubulin was used as a loading control.

In order to determine if the PP1β paralogs function independently or redundantly in vivo, we designed translation blocking morpholino antisense oligonucleotides to *ppp1cba* and *ppp1cbb*. The translation blocking morpholinos would be expected to knock down expression of both maternal and zygotic mRNA and were designed with no sequence overlap between the paralogs. These morpholinos were injected either individually or in combination into embryos at the one-cell stage, and the patterning and morphogenesis of the embryos was examined at the onset of gastrulation (50% epiboly and shield stage), just before (90% epiboly) or after blastopore closure (bud stage) or at 48 hpf. Injection of morpholinos targeted against either the *ppp1cba* (2.5 ng; 112/126 embryos) (Figure 7B) or *ppp1cbb* (2.5 ng; 121/145 embryos) (Figure 7C) alone resulted in a severe reduction in body axis elongation as assayed at 48 hpf and caused a dose dependent reduction in PP1β protein levels (Figure 7K) as determined using an antibody that recognizes both isoforms equally (Figure S2). The morphant embryos had a dramatically shortened and curved body axis with a dramatic reduction in the yolk extension and posterior structures. Higher doses of the morpholino (up to 10 ng for each morpholino) resulted in a delay, but not failure, of epiboly, a reduction in melanocyte pigmentation and a reduction in head and eye size. These higher dose phenotypes could not be readily rescued by mRNA co-injection and were not further characterized because they are difficult to distinguish from a morpholino artifact and are not present at the lower doses used through the rest of the manuscript. In addition, injection of a cocktail of both morpholinos (0.75 ng of *ppp1cba* and 0.75 ng of *ppp1cbb* MO, referred to as 2MO) resulted in a similar overall phenotype (Figure 7D; 144/171 embryos). Interestingly, the *ppp1cba/ppp1cbb* morphant phenotype resembles a severe *mypt1* morphant phenotype, which also displays a reduction of convergence and extension movement during gastrulation [9].

We next set out to determine if injection of mRNA from either *ppp1cba* or *ppp1cbb* would also disrupt body axis elongation. Embryos injected with 200 pg of either paralog did display a phenotype of a small head and eyes and a slight reduction in the darkness of the melanocytes but showed no significant defect in body axis elongation at 48 hpf (Figure 7 E and F; 111/112 Ba embryos, 105/105 Bb embryos). Co-injection of mRNA for either *ppp1cba* (84/105 embryos) or *ppp1cbb* (88/111 embryos) could rescue the shortened body axis phenotype of *ppp1cbb* morphants (Figure 7 G and H). Importantly the *ppp1cbb* morpholino was designed to the 5’ UTR of *ppp1cbb* and does not overlap significantly with the sequence of the expressed mRNA. In addition, *ppp1cbb* mRNA could rescue the *ppp1cba* morphant phenotype or the phenotype of a double knockdown embryo (Figure 7I; 79/106 embryos). A rescue experiment of either the *ppp1cba* MO or the 2MO cocktail using *ppp1cba* mRNA was not performed because this mRNA completely complements the *ppp1cba* morpholino, and would thereby complicate interpretation. Interestingly, severe defects in gastrulation occurred with the cocktail at lower doses (1.5 ng used for the rest of the manuscript) that only slightly reduced PP1β protein levels, but higher doses (3.0 ng) resulted in further knockdown of PP1β. Also, injection of single morpholinos resulted in a similar decrease in total PP1β levels, but rescue experiments where *ppp1cbb* mRNA is co-injected with the 2MO cocktail resulted in mild overexpression of PP1β(Figure 7K). Taken together these data provide strong evidence that these paralog function in an additive dose-dependent manner to assemble the myosin phosphatase in vivo. Any reduction in total PP1β protein results in a severe gastrulation defect, which can be rescued by *ppp1cb* mRNA injection.

Morpholinos are a valuable tool for studying gene function in zebrafish, but it is critically important to control for possible off-target effects and morpholino-induced cell death [53]. To control for morpholino toxicity we co-injected the p53 morpholino with the 2MO cocktail and an mRNA rescue. No significant difference in phenotype was observed between 2MO embryos co-injected with either the p53 morpholino (102 of 132 embryos had a severe gastrulation defect) or a control morpholino (100 of 120 embryos) (Figure S3). Furthermore, rescued embryos showed no significant difference between embryos co-injected with either the control morpholino (55 of 75 embryos were rescued) or the p53 morpholino (80 of 110 embryos were rescued). This was further demonstrated by injection of *ppp1cba* and *ppp1cbb* 5 base mismatch morpholinos. These mismatch morpholinos either alone or in a cocktail did not produce an observable phenotype when injected at the same doses used for the experimental morpholinos (99 of 110 ba-MM MO embryos; 85 of 91 bb-MM MO embryos; 115 of 121 2MO-MM embryos displayed no body axis defect) (Figure S4). Finally, the morpholino knockdown phenotype could be phenocopied with injection of alternate translation blocking morpholinos targeted to *ppp1cba* and *ppp1cbb* either alone (109 of 127 ba-MO-2 embryos and 144 of 157 bb-MO-2 embryos had severe gastrulation defects) or in combination (123 of 142 of 2MO-2 embryos had severe gastrulation defects) (Figure S4). Taken together these controls provide strong evidence that the observed body axis elongation defect is caused by specific knock-down of the PP1β paralogs.

**Figure 8:**
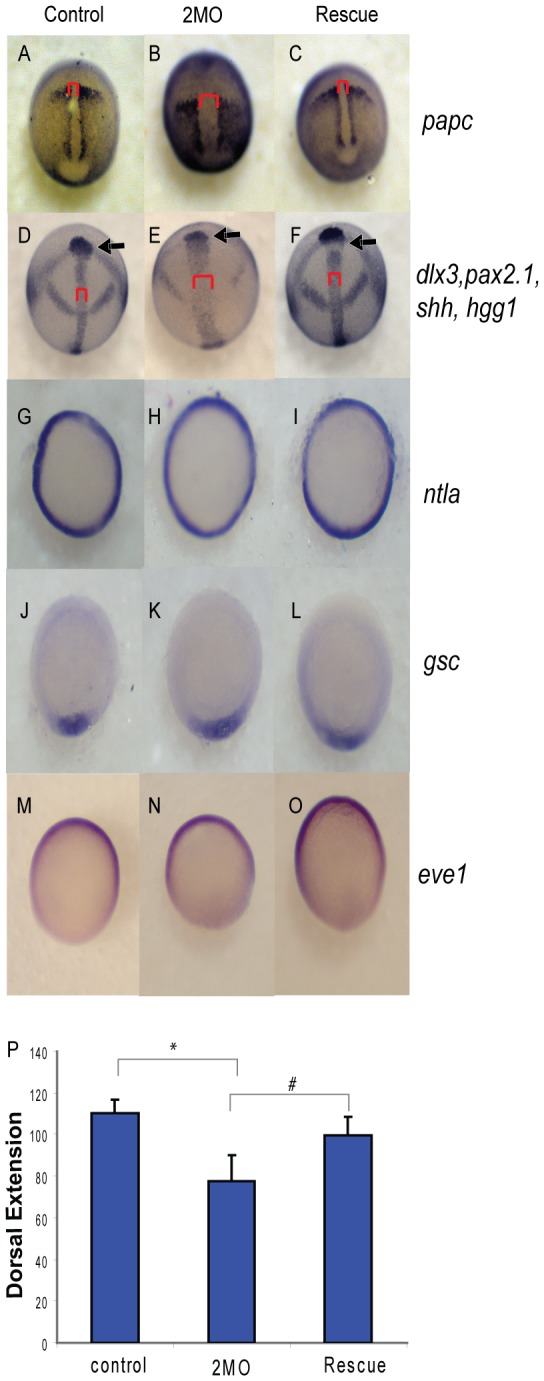
PP1β knockdown blocks convergence and extension but does not alter mesodermal cell fate or dorsal-ventral patterning. Embryos injected with either 1.5 ng of the 2 MO cocktail (0.75 ng *ppp1cba* MO and 0.75 ng of *ppp1cbb* MO) or 1.5 ng of the cocktail with 200 pg of *ppp1cbb* mRNA (rescue) were grown alongside uninjected clutch-mates (control) and staged and fixed for in situ hybridization. Embryos at 90% epiboly were fixed and stained with (A–C) *papc* (presomitic mesoderm) and imaged from an angle approximately 30 degrees from dorsal to allow visualization of the prechordal plate (marked with an arrow). Embryos at bud stage stained with *hgg1* (to mark the prechordal plate), *shh* (midline), *pax2.1* (midbrain-hindbrain boundary) and *dlx3* (neural plate) (D–F) and imaged with a view from dorsal. The red bracket marks the width of the notochord and the black arrow points to the prechordal plate. Embryos at 50% epiboly were stained with *ntla* (G–I) to assay for mesoderm induction, *gsc* to assay for dorsal cell fates (J–L) and *eve1* (M, N, O) for ventral cell fates, all viewed from the animal pole, with dorsal facing down. (P) Quantification of body axis elongation at bud stage for embryos injected with the indicated reagents. Each injection experiment was performed at least 3 times and the morphogenetic measurements were performed on 50 48 hpf and 50 bud stage embryos. Error bars are standard error and a black * indicates a statistically significant difference from control and a # indicates a statistically significant rescue compared to morpholino injected embryos. Statistical significance was calculated using a one-factor ANOVA with Tukey post hoc analysis and is defined as p < 0.05.

Defects in body axis elongation can be caused by several underlying phenotypes; thus we set out to directly assay morphogenesis and patterning during gastrulation. Control, 2MO or rescued embryos (2MO plus *ppp1cbb* mRNA) were fixed and subjected to in situ hybridization at the indicated stages (Figure 8). The presomitic mesodermal marker *papc* (also called *pcdh8*) was used to directly determine the length and width of both the notochord and the presomitic mesoderm in embryos at 90% epiboly just before blastopore closure. Morphant embryos (Figure 8B, 39/48 embryos) displayed a dramatically broader and shorter notochord (red brackets) and a broader and shorter presomitic mesodermal domain than control embryos (Figure 8A, 52/53 embryos). Rescued embryos displayed a phenotype resembling the control embryos with a normal width notochordal and presomitic mesoderm (Figure 8C, 32/41 embryos). To complement the *papc* assay a cocktail of four in situ probes was used to characterize any convergent extension defect in bud-staged embryos containing (*shh* - to mark the notochord, *hgg1* - to mark the prechordal plate, *pax2.1* - to mark the midbrain hindbrain boundary (also called *pax2a*); and *dlx3* - to mark the neural plate. Morphant embryos displayed severely shortened and broadened notochordal (red brackets) and presomitic mesodermal tissue (blue brackets) (Figure 8 E, 51/65 embryos) compared to control (Figure 8D 56/56 embryos). In addition, the neural epithelium (as indicated by the breath of *pax2.1* staining) and anterior migration of the prechordal plate was reduced (Figure 8 E). The rescued embryos displayed approximately normal morphogenesis with the anterior migration of the prechordal plate being rescued to a lesser extent than presomitic mesodermal convergence and extension (71/86 embryos). Interestingly, the 2MO embryos display a gastrulation phenotype that closely resembles, but is slightly more severe than *mypt1* MO embryos [9].

To provide a quantitative measure of convergence and extension at the end of gastrulation, lateral views of 50 cocktail-stained embryos were imaged, and the angle between the leading edge of the prechordal plate and the end of the notochord was measured and used as an indicator of body axis elongation (Figure 8P). Morphant embryos had significantly reduced body axis elongation, while the rescued embryos more closely resembled control embryos (Figure 8P). It is possible that defects in mesodermal morphogenesis are secondary to defects in mesodermal induction or dorsal-ventral axis formation, thus we set out to directly assay for patterning defects. Morphant (36/36), control (42/42) and rescued (61/61) embryos all displayed a normal pattern of *ntla* (a marker of mesendoderm) at 50% epiboly, indicating that mesodermal induction is unaffected by *ppp1cba/ppp1cbb* knockdown (Figure 8 G, H and I). In addition the dorsal marker *gsc* was normal in morphant (37/41), rescued (45/45) and control embryos (48/48), indicating no disruption of dorsal cell fates. Ventral cell fates also appeared normal in morphant (30/31), control (44/44) and rescued embryos (48/48) as assayed by *eve1* (Figure 8 M, N, O) or *bmp4* (data not shown). Taken together these data provide strong evidence that *ppp1cba/ppp1cbb* morphant embryos have broadly normal mesodermal induction and dorsal ventral patterning, but have serious defects in convergence and extension of the presomitic mesoderm and anterior migration of the prechordal plate.

**Figure 9:**
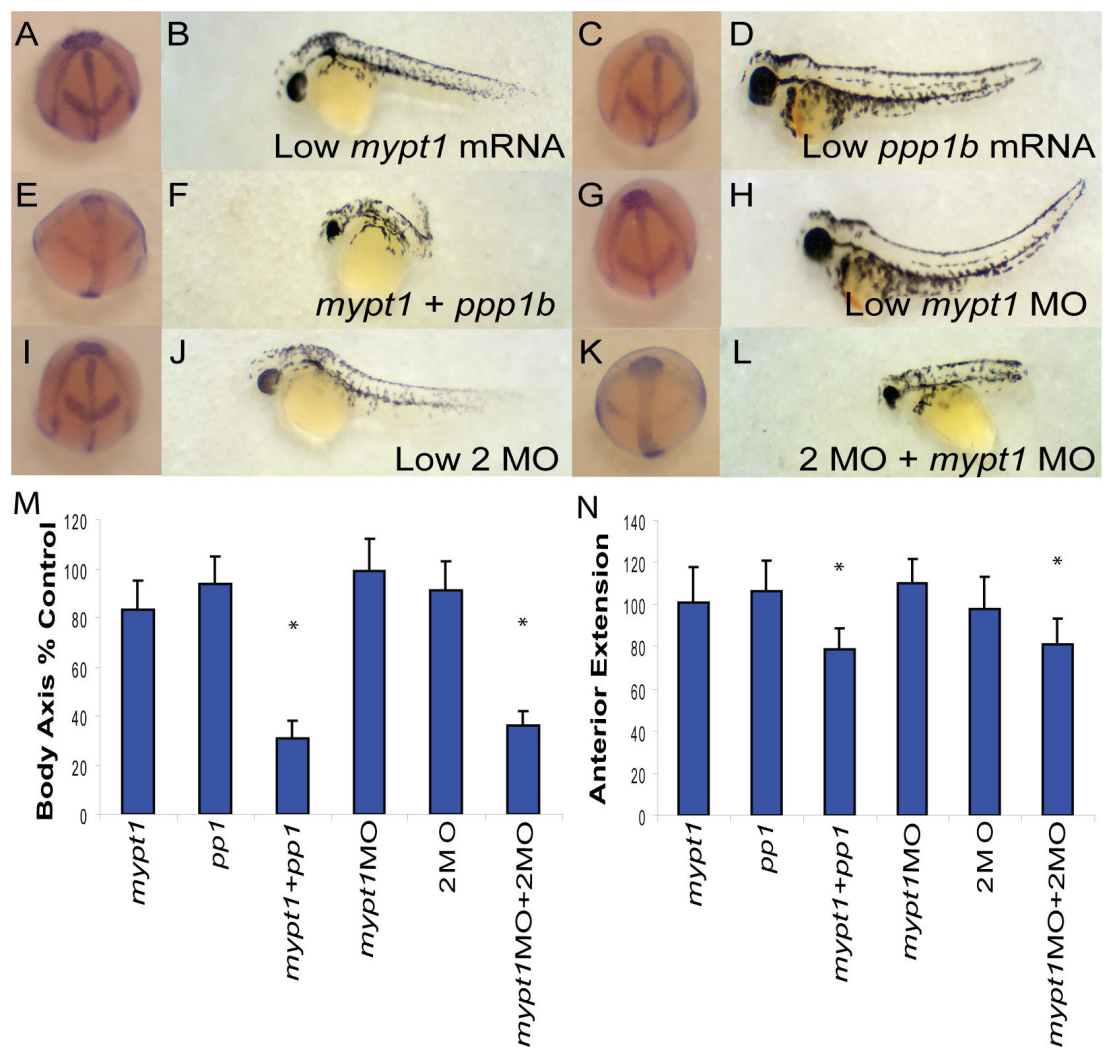
PP1β genes interact genetically with Mypt1. (A–L) Representative embryos at bud stage (A, C, E, G, I, K) and 48 hpf (B, D, F, H, J, L) injected with 15 pg *mypt1* (1-300) mRNA with 100 pg of GFP mRNA (A–B), 50 pg each of *ppp1cba* and *ppp1cbb* mRNA with 15 pg GFP mRNA (C–D), 15 pg *mypt1* mRNA + 50 pg each of *ppp1cba* and *ppp1cbb* mRNA (E–F), 0.5 ng of *mypt1* MO and 0.25 ng of the control MO (G–H), 0.5 ng control MO and 0.25 ng 2MO (I–J), 0.5 ng *mypt1* MO and 0.25 ng 2MO (0.125 ng *ppp1cba* MO and 0.125 ng of *ppp1cbb* MO) (K–L). Quantification of the angle of body axis extension at bud stage (M) and 48 hpf (N). Error bars are standard error and a * indicates a statistically significant difference from control and a # indicates a statistically significant rescue compared to morpholino injected embryos. For statistical analysis 50 embryos were analyzed for each condition. Statistical significance was calculated using a one-factor ANOVA with Tukey post hoc analysis and is defined as p < 0.05.

Given the similarity of phenotypes observed between *ppp1cba/ppp1cbb* 2MO embryos and *mypt1* MO embryos, we set out to directly determine if the PP1β paralogs genetically interact with *mypt1*. Injection of suboptimal doses of the 2MO cocktail (Figure 9 I-J; 55/57 bud, 62/66 48 hpf embryos) or *mypt1* morpholino (Figure 9 G-H; 61/66 bud, 55/61 48 hpf embryos) did not cause a significant defect in body axis elongation at either bud stage or 48 hpf. However, combining the morpholinos resulted in a severe defect in gastrulation (Figure 9 K-L; 42/60 bud, 43/61 48 hpf embryos), indicating that these genes cooperate to produce an active myosin phosphatase. Total morpholino and mRNA doses were balanced using the Gene Tools control morpholino and GFP mRNA. In addition, mRNA co-injection experiments were performed with a *mypt1* mRNA that encodes the first 300 amino acids in Mypt1 (Figure 9 A-B) and a 50/50 mix of *ppp1cba/ppp1cbb* mRNA (Figure 9 C-D). As in Figure 7, over-expression of PP1β did not significantly disrupt gastrulation (61/61 bud, 62/66 48 hpf embryos). *Mypt1* mRNA results in a severe gastrulation defect at higher doses (150 pg – data not shown) but a low dose of 15 pg was used, which results in little disturbance of body axis elongation (67/67 bud, 55/57 48 hpf). Co-injection of *mypt1* and *ppp1cba/ppp1cbb* mRNA resulted in a severe defect in gastrulation (Figure 9 E-F; 51/62 bud, 85/111 48 hpf embryos), consistent with the formation of a constitutively active complex. Body axis elongation at both bud stage and 48 hpf was quantified (Figure 9 M-N).

**Figure 10:**
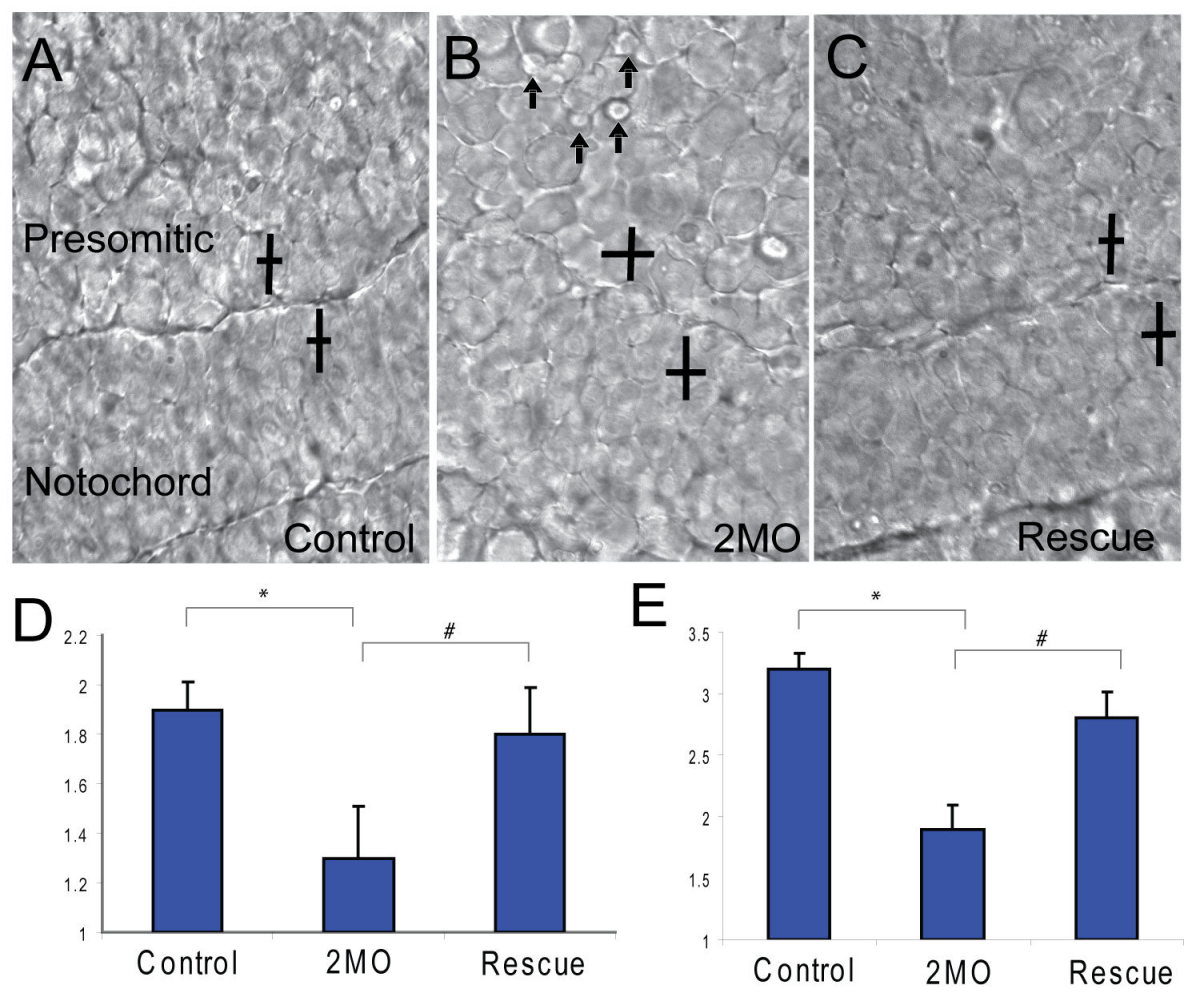
PP1β paralogs are required for cell shape changes required for convergent extension. A representative field of presomitic and notochordal mesoderm at the bud stage in control (A), 2MO (0.75 ng *ppp1cba* MO and 0.75 ng of *ppp1cbb* MO) embryos (B) or rescued embryos (C). The cell polarity of presomitic (D) and notochordal (E) mesodermal cells was determined by calculating the length width ratio (y-axis). The crossed bars indicate cells with the long axis length and the short axis width. The embryos are arranged such that dorsal is down and anterior is to the right. Arrows indicate cells that are actively producing bleb-like protrusions. Error bars are standard error and a * indicates a statistically significant difference from control. All calculations were made on 75 cells from 3–5 separate embryos. Statistical significance was calculated using a one-factor ANOVA with Tukey post hoc analysis and is defined as p < 0.05.

Cell intercalation is one of the primary mechanisms of mesodermal convergent extension in zebrafish [54]. Mypt1 is required for proper cell elongation of mesodermal cells as they undergo intercalation [9]. Thus, we set out to determine if mesodermal cell morphology was missregulated in 2MO cocktail morphant embryos. We calculated the length-width ratios of mesodermal cells at the bud stage in control embryos compared to embryos injected with the 2MO cocktail and rescued embryos (Figure 10 A–C). Control mesodermal cells are highly polarized and elongated at bud stage in both presomitic and axial mesoderm (Figure 10 D and E). Both presomitic and notochordal mesodermal cells are significantly less elongated in morphant embryos, with rescued embryos being similar to control embryos (Figure 10D and E). Interestingly, morphant mesodermal cells display excessive bleb-like membrane protrusions and a disruption in cell packing similar to the phenotype observed in Mypt1 morphants [9] (Figure 10B). These results clearly indicate that loss of *ppp1cba/cbb* results in cells that are less elongated than control cells and display increased membrane blebbing.

## Discussion

This study provides the first functional characterization of the two catalytic subunits of the zebrafish myosin phosphatase. We observed that zebrafish has two paralogs that encode proteins homologous to the β isoform of PP1 termed PP1Ba and PP1Bb. Both genes were maternally and zygotically expressed throughout early zebrafish development and displayed broad expression patterns. We further observed that both PP1Ba and PP1Bb could interact directly with Mypt1 in GST pull down and co-IP experiments with similar affinities. Mutating the PP1 binding motif in Mypt1 from KVKF to KMKF, as found in the zebrafish mutant allele *sq181*, resulted in a loss of affinity in IP experiments, but some residual binding was still observed. In contrast, mutating the domain to KAKA abrogated binding. We next carried out functional experiments with PP1Ba and PP1Bb and found that they could assemble an active myosin phosphatase complex in HeLa cells. Cells expressing WT Mypt1 and PP1Ba or PP1Bb displayed dramatic rearrangements of the actin cytoskeleton, reduced MLC2 phosphorylation and had collapsed neural-like cell morphology.

In vivo, morpholino knockdown of either or both paralogs results in a severe defect in body axis elongation due to failure of proper mesodermal convergent extension. Importantly, precise dosage of the gene products is required for early development. A modest reduction in total PP1β protein results in severe gastrulation defects, but these defects can be rescued by co-injecting mRNA encoding either isoform. This provides direct evidence that the paralogs function similarly during gastrulation. Interestingly, the endogenous genes appear to be unable to complement for the loss of the other paralog, most likely because of dose dependency. Injection of mRNA, in contrast, can complement for the knock-down of the other paralog by producing an overexpression of PP1β. The dose-dependency model is further supported by the fact that even modest knockdown of *mypt1* results in a severe gastrulation phenotype [9]. PP1β morphants displayed a phenotype highly similar to *mypt1* morphants, and interacted genetically with either increased *mypt1* expression or *mypt1* knockdown. These observations provide strong evidence that PP1Ba and PP1Bb function to assemble the myosin phosphatase in vivo.

Duplication of genes in zebrafish is a common phenomenon due to a genome duplication early in the evolution of teleosts [55]. The amino acid sequence and expression patterns of *ppp1cba* and *ppp1cbb* have diverged very little during zebrafish evolution, indicating selective pressure to maintain both paralogs. In our genetic and biochemical experiments both paralogs appeared to act essentially identically. It is therefore surprising that both paralogs would be maintained. Morpholino knockdown of either paralog results in a severe defect during gastrulation, which may explain this conservation of function. If precise gene dosage is required for proper myosin phosphatase function in early development, it may have proven difficult for the functions of the paralogs to diverge. Indeed minor perturbations of myosin phosphatase activity can result in severe developmental defects [9].

In our work we chose to focus on convergence and extension defects, which are the first identifiable phenotype in embryos with reduced myosin phosphatase activity [9]. CE cell movements are controlled by a number of signaling pathways including Bmp signaling [56,57], Eph-ephrin signaling [58], PDGF-PI3K [59,60], Matrix-metalloproteases [61], Septins [62] and Jak-Stat signaling [63,64]. The Wnt-Planar cell polarity (Wnt-PCP) pathway is, however, the most widely studied regulatory pathway [26–28,65]. The Wnt-PCP pathway activates several downstream targets including the small GTPases RhoA and Rac1, which mediate many Wnt-dependent effects on the cytoskeleton [30,66,67], such as cellular contractility, polarity and adhesion [26,27]. The Wnt-PCP pathway requires finely balanced regulation to elicit polarize cell movement as either gain or loss-of-function of Wnt-PCP components results in severe gastrulation defects [68–75]. RhoA is controlled by several additional regulators during zebrafish gastrulation including the non-receptor tyrosine kinases Fyn and Yes [76,77], which signal along with tyrosine phosphatases Shp2 [78], RPTPα and PTPε [79,80] In addition pathways dependent on Gα12 and Gα13 [81], AKAP12 (Gravin) [29,82] Adhesion-associated GAPs [83] and several Rho-GEFs [84–87] control RhoA signaling and disruption of any of these regulators causes severe gastrulation and body axis elongation defects. Critical targets of RhoA during gastrulation include formins [88], which control actin polymerization, and the Rho-dependent kinase Rock [30,89] and myosin phosphatase [9], which control actomyosin contractility [20]. Much like the Wnt-PCP pathway perturbations that cause increased or decreased contractility lead to severe CE defects [9,29,30]. Genetic evidence clearly demonstrates that Rock and Mypt1 are critical downstream targets of the Wnt-PCP pathway during fish gastrulation [9,30]. Rock-mediated phosphorylation of Mypt1 causes inhibition of the bound PP1 which leads to increased MLC2 phosphorylation [9]. The phenotypic similarity between our *ppp1cba*/*ppp1cbb* knockdown and *mypt1* knockdown indicates that the essential role of PP1β in early development is assembly of the myosin phosphatase and implicates PP1β in the molecular regulation of cell motility during gastrulation.

While we observed an overlapping role for *ppp1cba* and *ppp1cbb* during gastrulation, we cannot exclude the possibility that the paralogs have diverged in function during later developmental processes that involve Mypt1 such as neural epithelial relaxation and liver development [18,19]. A zygotic role for *ppp1cba* has been examined but no characterization of *ppp1cbb* has been performed; thus we cannot determine their genetic relationship [18]. It would be interesting to examine the role of both paralogs in these later developmental processes perhaps using splice-blocking morpholinos, which will only disrupt expression of zygotic mRNA. In most of our experiments we observed that loss-of-PP1β function was similar to, but more severe than loss-of-Mypt1 function [9]. This phenomenon could be due to a number of factors such as differences in protein stability or maternal protein deposits. In addition, PP1β may function through other holoenzyme complexes containing regulatory subunits other than Mypt1. We cannot exclude a role for other related targeting subunits such as Mypt2, Mypt3 and TIMAP (reviewed in 5) or yet other unidentified targeting subunits, and a further analysis of PP1 binding proteins in early development may be warranted. Additionally, given the considerable functional redundancy between PP1 isoforms observed in other organisms [15,33–35], it could be quite informative to characterize overlapping functions with other zebrafish PP1 genes such as *ppp1caa*, *ppp1cab* and *ppp1cc*. Such an analysis may uncover additional roles for *ppp1cba* and *ppp1cbb*.

In conclusion, these data provide direct evidence that zebrafish have two widely expressed catalytic subunits for the myosin phosphatase. This study will provide valuable insight into the role of the myosin phosphatase in complex cellular and developmental behaviors such as gastrulation, neural epithelial biology and liver development. This study also indicates that zebrafish may be a valuable model for characterizing the complex regulation and evolution of PP1 genes.

## Supporting Information

Figure S1 **Mutation of the PP1-binding domain of Mypt1 reduces the ability to assemble an active myosin phosphatase complex.** HeLa cells were transfected with either KMKF Mypt1 and GFP (A, B), KAKA Mypt1 and GFP (C, D), KMKF Mypt1 and PP1Ba (E, F), KMKF Mypt1 and PP1Bb (G, H), KAKA Mypt1 and PP1Ba (I, J), KAKA Mypt1 and PP1Bb (K, L). All cells were fixed, and stained with DAPI and Alexa 568-phalloidin and imaged with confocal microscopy. Black and white images show phalloidin staining, while color images are a merge of DAPI (blue), GFP (green) and phalloidin (red).
(TIF)

Figure S2 **The gene products of *ppp1cba* and *ppp1cbb* are both recognized by PP1β antibodies.** HEK293T cells were either mock-transfected or transfected with GFP-tagged PP1Ba, GFP-tagged PP1Bb or GFP-tagged mammalian PP1β. The cells were lysed, run on SDS-Page and blotted with anti-GFP or anti-PP1β antibodies.
(TIF)

Figure S3 **The *ppp1cba/ppp1cbb* knockdown phenotype is independent of p53.** (A–C) Lateral views of representative 48 hpf zebrafish embryos injected with (A) 4 ng p53 MO, (B) 0.75 ng *ppp1cba* MO, 0.75 ng of *ppp1cbb* MO (2MO) and 4 ng p53 MO (C) a partially rescued embryo injected with 100 pg *ppp1cbb* mRNA, 0.75 ng of *ppp1cbb*, 0.75 *ppp1cba* MO and 4 ng p53 MO. (D) In addition, control embryos were injected with 4 ng control MO; 2MO and 4 ng control or 2MO, 100 pg *ppp1cbb* mRNA and 4 ng of control MO (D) Quantification of the truncated body axis phenotype in morphant and mRNA injected embryos. Each injection was performed multiple times with 25 embryos used to calculate body axis length and reported as % of uninjected clutch mates. Error bars are standard error, a black * indicates a statistically significant difference from control, # indicates a statistically significant rescue and a NS indicates two data sets that are not significantly different. Statistical significance was calculated using a one-factor ANOVA with Tukey post hoc analysis and is defined as p < 0.05.
(TIF)

Figure S4 **Body axis elongation defects are induced by alternative *ppp1cba* and *ppp1cbb* morpholinos but not mismatched controls.** (A–C) Lateral views of representative 48 hpf zebrafish embryos injected with (A) 2.5 ng mismatch *ppp1cba* MO, (B) 2.5 ng mismatch *ppp1cbb* MO, (C) a mixture of 0.75 ng mismatch *ppp1cba* MO and 0.75 ng of mismatch *ppp1cbb* MO (2MO-MM), (D) 5.0 ng *ppp1cba* MO-2, (E) 5.0 ng *ppp1cbb* MO-2, (F) a mixture of 1.5 ng *ppp1cba* MO and 1.5 ng of *ppp1cbb* MO (2MO-2). (G) Quantification of the truncated body axis phenotype in morphant and control embryos. Each injection was performed multiple times with 25 embryos used to calculate body axis length and reported as % of uninjected clutch mates. Error bars are standard error and a black * indicates a statistically significant difference from control. Statistical significance was calculated using a one-factor ANOVA with Tukey post hoc analysis and is defined as p < 0.05.
(TIF)
