# Supplementary figures and images for: Protein Phosphatase 1 β Paralogs Encode the Zebrafish Myosin Phosphatase Catalytic Subunit
Source: PLoS One. 2013 Sep 11;8(9):e75766. doi: 10.1371/journal.pone.0075766 (PMC3770619; doi:10.1371/journal.pone.0075766)

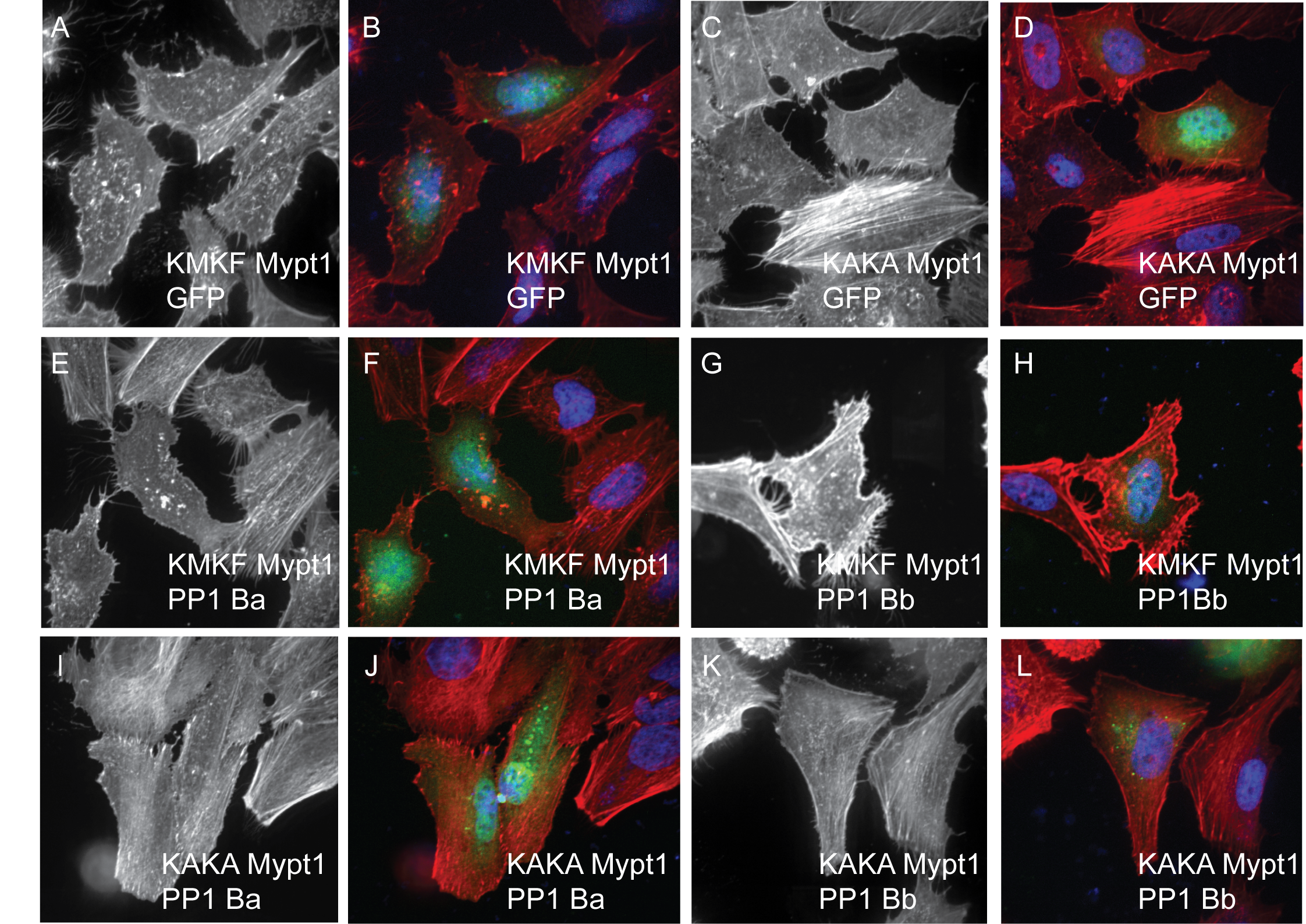

Supplement: Figure S1 — Mutation of the PP1-binding domain of Mypt1 reduces the ability to assemble an active myosin phosphatase complex. HeLa cells were transfected with either KMKF Mypt1 and GFP (A, B), KAKA Mypt1 and GFP (C, D), KMKF Mypt1 and PP1Ba (E, F), KMKF Mypt1 and PP1Bb (G, H), KAKA Mypt1 and PP1Ba (I, J), KAKA Mypt1 and PP1Bb (K, L). All cells were fixed, and stained with DAPI and Alexa 568-phalloidin and imaged with confocal microscopy. Black and white images show phalloidin staining, while color images are a merge of DAPI (blue), GFP (green) and phalloidin (red). (TIF) [file pone.0075766.s001.tif]

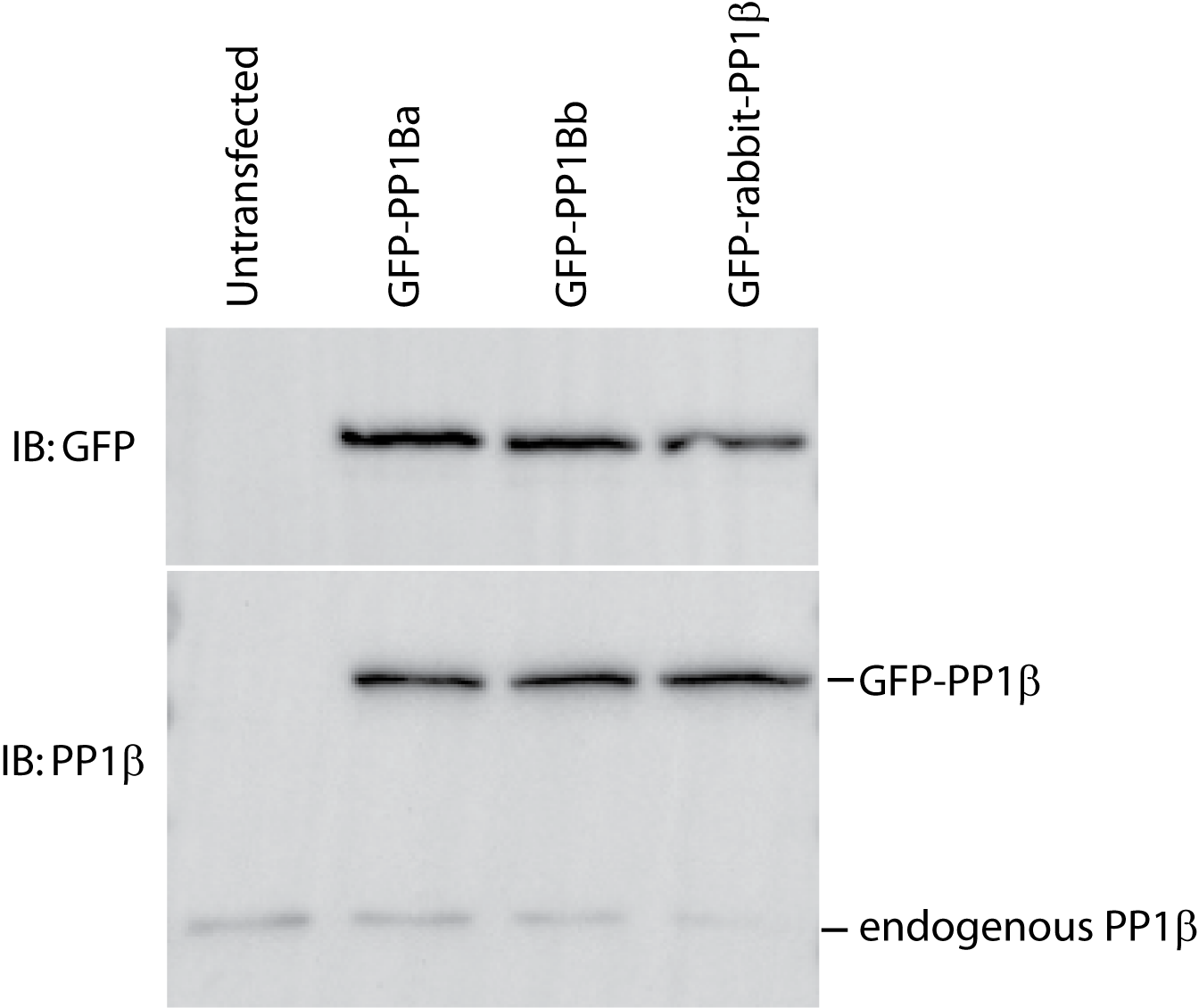

Supplement: Figure S2 — The gene products of ppp1cba and ppp1cbb are both recognized by PP1β antibodies. HEK293T cells were either mock-transfected or transfected with GFP-tagged PP1Ba, GFP-tagged PP1Bb or GFP-tagged mammalian PP1β. The cells were lysed, run on SDS-Page and blotted with anti-GFP or anti-PP1β antibodies. (TIF) [file pone.0075766.s002.tif]

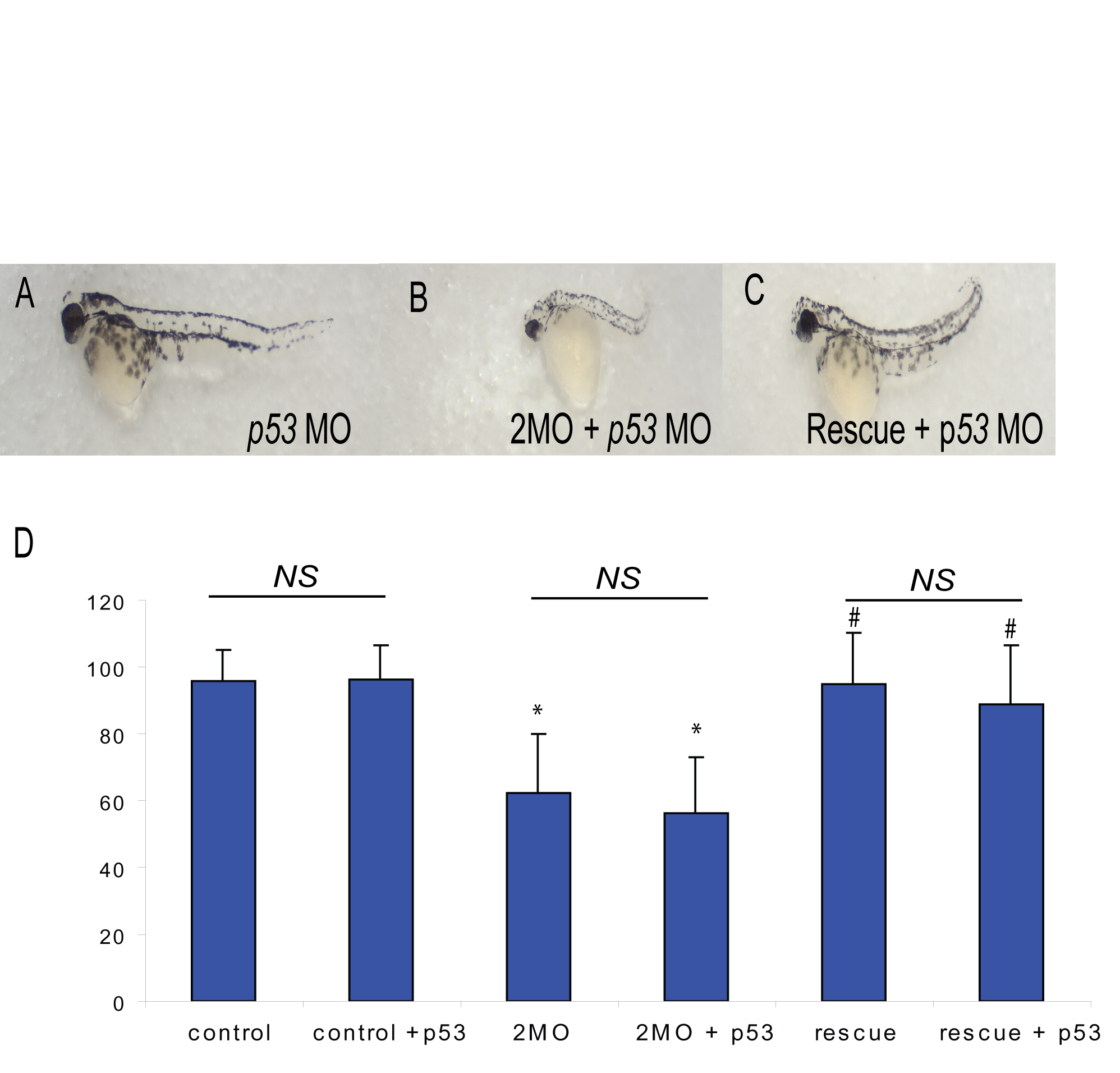

Supplement: Figure S3 — The ppp1cba/ppp1cbb knockdown phenotype is independent of p53. (A–C) Lateral views of representative 48 hpf zebrafish embryos injected with (A) 4 ng p53 MO, (B) 0.75 ng ppp1cba MO, 0.75 ng of ppp1cbb MO (2MO) and 4 ng p53 MO (C) a partially rescued embryo injected with 100 pg ppp1cbb mRNA, 0.75 ng of ppp1cbb, 0.75 ppp1cba MO and 4 ng p53 MO. (D) In addition, control embryos were injected with 4 ng control MO; 2MO and 4 ng control or 2MO, 100 pg ppp1cbb mRNA and 4 ng of control MO (D) Quantification of the truncated body axis phenotype in morphant and mRNA injected embryos. Each injection was performed multiple times with 25 embryos used to calculate body axis length and reported as % of uninjected clutch mates. Error bars are standard error, a black * indicates a statistically significant difference from control, # indicates a statistically significant rescue and a NS indicates two data sets that are not significantly different. Statistical significance was calculated using a one-factor ANOVA with Tukey post hoc analysis and is defined as p < 0.05. (TIF) [file pone.0075766.s003.tif]

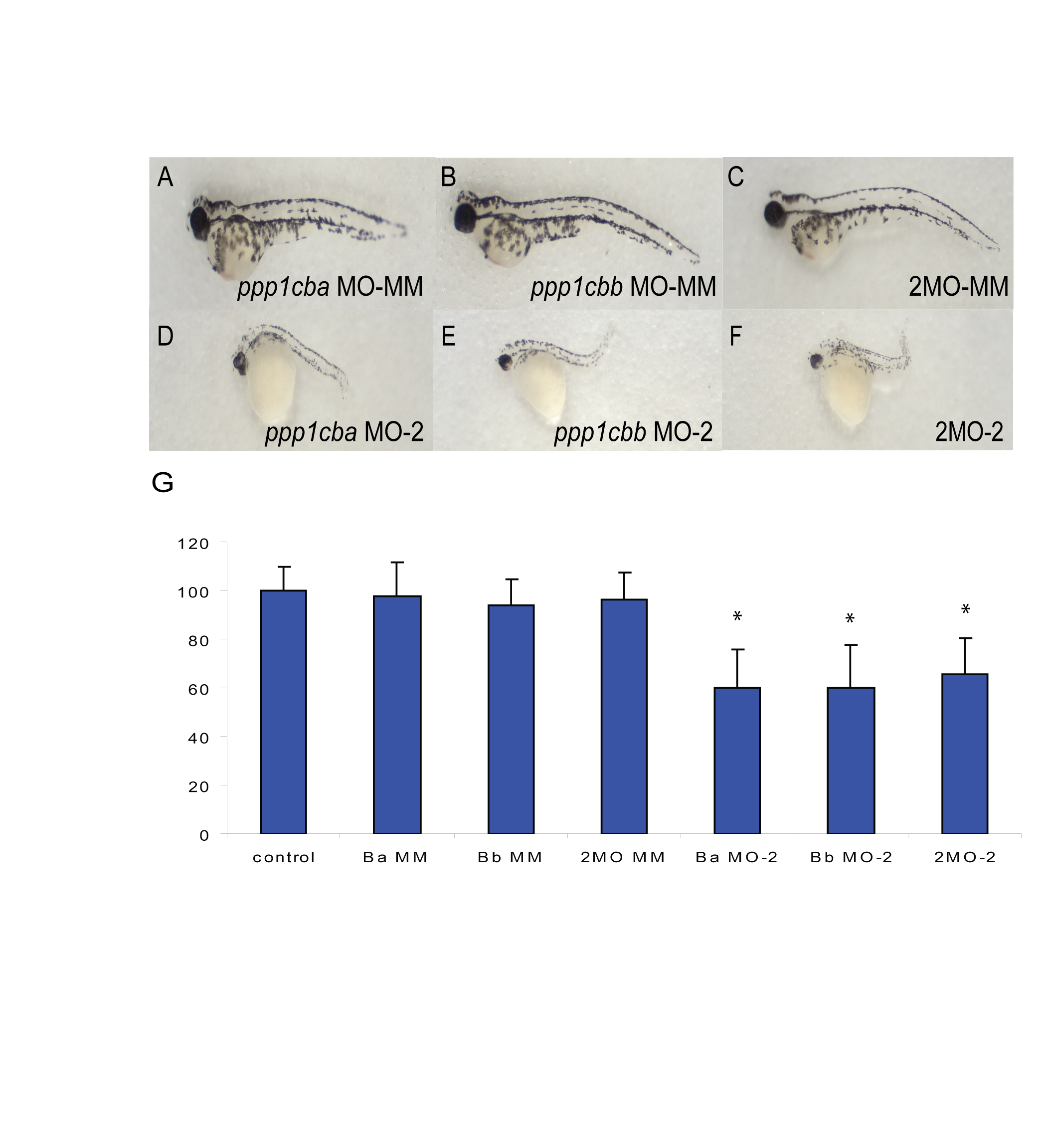

Supplement: Figure S4 — Body axis elongation defects are induced by alternative ppp1cba and ppp1cbb morpholinos but not mismatched controls. (A–C) Lateral views of representative 48 hpf zebrafish embryos injected with (A) 2.5 ng mismatch ppp1cba MO, (B) 2.5 ng mismatch ppp1cbb MO, (C) a mixture of 0.75 ng mismatch ppp1cba MO and 0.75 ng of mismatch ppp1cbb MO (2MO-MM), (D) 5.0 ng ppp1cba MO-2, (E) 5.0 ng ppp1cbb MO-2, (F) a mixture of 1.5 ng ppp1cba MO and 1.5 ng of ppp1cbb MO (2MO-2). (G) Quantification of the truncated body axis phenotype in morphant and control embryos. Each injection was performed multiple times with 25 embryos used to calculate body axis length and reported as % of uninjected clutch mates. Error bars are standard error and a black * indicates a statistically significant difference from control. Statistical significance was calculated using a one-factor ANOVA with Tukey post hoc analysis and is defined as p < 0.05. (TIF) [file pone.0075766.s004.tif]
